# Unveiling unique protein and phosphorylation signatures in lung adenocarcinomas with and without *ALK*, *EGFR*, and *KRAS* genetic alterations

**DOI:** 10.1002/1878-0261.70091

**Published:** 2025-07-07

**Authors:** Fanni Bugyi, Mirjam Balbisi, Simon Sugár, Lóránd Váncza, Eszter Regős, Ilona Kovalszky, Ibolya Laczó, Tünde Harkó, Gábor Kecskeméti, Zoltán Szabó, Judit Moldvay, László Drahos, Lilla Turiák

**Affiliations:** ^1^ MTA‐HUN‐REN TTK Lendület (Momentum) Glycan Biomarker Research Group, HUN‐REN Research Centre for Natural Sciences Budapest Hungary; ^2^ Hevesy György PhD School of Chemistry ELTE Eötvös Loránd University Budapest Hungary; ^3^ Semmelweis University Doctoral School Budapest Hungary; ^4^ Department of Pathology and Experimental Cancer Research Semmelweis University Budapest Hungary; ^5^ Békés County Central Hospital Gyula Hungary; ^6^ National Korányi Institute of Pulmonology Budapest Hungary; ^7^ Department of Medical Chemistry, Albert Szent‐Györgyi Medical School University of Szeged Hungary; ^8^ Pulmonology Clinic, Albert Szent‐Györgyi Medical School University of Szeged Hungary; ^9^ MS Proteomics Research Group, HUN‐REN Research Centre for Natural Sciences Budapest Hungary

**Keywords:** cancer research, FFPE tissue, genetic alterations, lung adenocarcinoma, mass spectrometry, phosphoproteomics

## Abstract

Genetic alterations in key oncogenes have been frequently identified in lung adenocarcinoma (LUAD), including genes encoding epidermal growth factor receptor (*EGFR*), Kirsten rat sarcoma viral oncogene homolog (*KRAS*), and anaplastic lymphoma kinase (*ALK*). In this pilot study, we aimed to characterize the differences in enriched biological pathways and phosphorylation events between LUAD tumors harboring *EGFR*, *KRAS*, or echinoderm microtubule‐associated protein‐like 4 (*EML4*)–*ALK* oncogenic alterations and triple wild‐type LUAD tumors (WT, without *EML4–ALK*, *KRAS*, or *EGFR* alterations) by mass spectrometry (MS)‐based quantitative proteomics and phosphoproteomics. We analyzed tumor regions of 82 formalin‐fixed paraffin‐embedded (FFPE) tissue sections with 6, 23, 31, and 22 samples from the *EML4–ALK*, *EGFR*, *KRAS*, and WT sample groups, respectively. A total of 1377 to 2189 proteins and 73 to 1781 phosphosites were quantified in these analyses. Based on the results, the samples clustered according to their genetic alteration type, and *EGFR*‐mutated samples showed unique protein expression patterns. Membrane organization, vesicle organization, and vesicle‐mediated transport Gene Ontology Biological Process (GOBP) terms were significantly downregulated in *EGFR*‐mutated samples compared to the other sample groups. Changes in 36 proteins and 52 phosphosites were also identified as potentially specific to a given genetic alteration. Many of these proteins have previously been linked to *EGFR* or *KRAS* mutations [e.g., cathepsin L, stimulator of interferon genes protein (STING)], whereas several phosphoproteins are associated with RNA splicing [e.g., serine/arginine‐rich splicing factor 1 (SRSF1), SRSF2, and SRSF7 proteins]. Kinase–substrate enrichment analysis indicated altered activities of 10 kinases, including mitogen‐activated protein kinases (MAPKs) and cyclin‐dependent kinases (CDKs). For example, CDK2 activity was elevated in *EML4–ALK* samples compared to the other sample groups. Our results could provide significant insights into further studies that could contribute to developing improved diagnostic and therapeutic strategies for LUAD.

## Graphical abstract

Proteomic and phosphoproteomic analyses were performed on lung adenocarcinoma (LUAD) tumors with *EGFR*, *KRAS*, or *EML4–ALK* alterations and wild‐type cases. Distinct protein expression and phosphorylation patterns were identified, especially in *EGFR*‐mutated tumors. Key altered pathways included vesicle transport and RNA splicing. The findings highlight molecular differences that could support the refinement of LUAD classification and guide future therapeutic development.

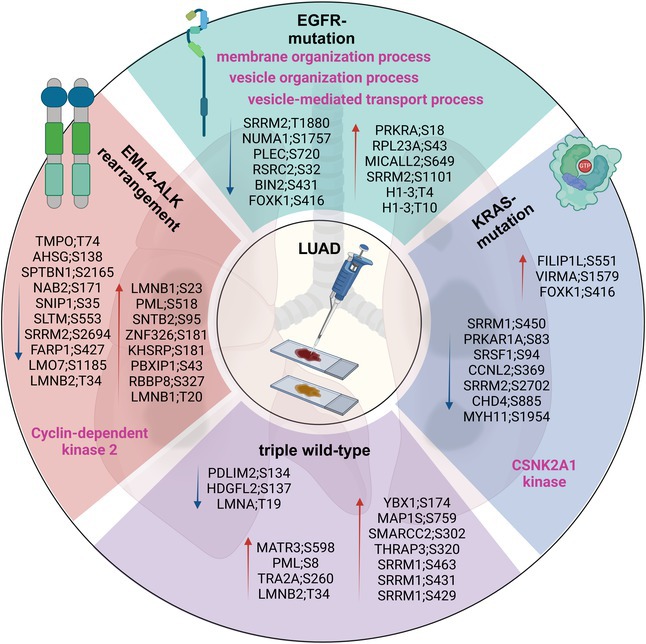

**Abbreviations**
ABC: ammonium‐bicarbonate
ACN: acetonitrile
ALK: anaplastic lymphoma kinase
ANOVA: analysis of variance
BP: biological process
CATL1: procathepsin L
CDK1: cyclin‐dependent kinase 1
CDK2: cyclin‐dependent kinase 2
CDKs: cyclin‐dependent kinases
CRIP1: cysteine‐rich protein 1
CSNK2A1: casein kinase 2 alpha 1
DDA: data‐dependent analysis
EGFR: epidermal growth factor receptor
EML4: echinoderm microtubule‐associated protein‐like 4
ERBB2: receptor tyrosine‐protein kinase erbB‐2
FA: formic acid
FDA: Food and Drug Administration
FDR: false discovery rate
FFPE: formalin‐fixed paraffin‐embedded
FISH: fluorescence *in situ* hybridization
GO: Gene Ontology
GOBP: Gene Ontology Biological Process
GOCC: Gene Ontology Cellular Component
GOMF: Gene Ontology Molecular Function
GSEA: gene set enrichment analysis
IHC: immunohistochemistry
KRAS: Kirsten rat sarcoma viral oncogene homolog
KSEA: kinase–substrate enrichment analysis
LAMP1: lysosome‐associated membrane glycoprotein 1
LC: lung cancer
LUAD: lung adenocarcinoma
MAPK1: mitogen‐activated protein kinase 1
MAPK11: mitogen‐activated protein kinase 11
MAPK7: mitogen‐activated protein kinase 7
MAPKs: mitogen‐activated protein kinases
MS: mass spectrometry
NES: normalized enrichment score
NSCLC: non‐small cell lung cancer
PCR: polymerase chain reaction
PGDH: 15‐hydroxyprostaglandin dehydrogenase
PPI: protein–protein interaction
PSPB: pulmonary surfactant‐associated protein B
SCAMP2: secretory carrier‐associated membrane protein 2
SCLC: small‐cell lung cancer
SRFS1: serine/arginine‐rich splicing factor 1
SRFS2: serine/arginine‐rich splicing factor 2
SRFS7: serine/arginine‐rich splicing factor 7
SRMM1: serine/arginine repetitive matrix protein 1
SRMM2: serine/arginine repetitive matrix protein 2
STING: stimulator of interferon genes protein
TFA: trifluoroacetic acid
UMAP: uniform manifold approximation and projection
WT: triple wild‐type

## 1 Introduction

Lung cancer (LC) is the leading cause of cancer‐related mortality, accounting for 11.4% of new cancer incidences in 2020 based on the Global Cancer Statistics [1, 2]. LC is histologically classified into small cell lung cancer (SCLC, 15% of all LC) and non‐small cell lung cancer (NSCLC, 85% of all LC). The main subtypes of NSCLC are adenocarcinoma, squamous cell carcinoma, and large cell carcinoma. Lung adenocarcinoma (LUAD) accounts for approximately 40% of all LC cases [3].

Genetic alterations in key oncogenes have been frequently identified in LUAD, including genes encoding epidermal growth factor receptor (*EGFR*), Kirsten rat sarcoma viral oncogene homolog (*KRAS*), and anaplastic lymphoma kinase (*ALK*) [4, 5]. These genetic variations are specific molecular targets for therapeutic intervention and provide prognostic value [6, 7].

Mutations in *EGFR* occur in 30–40% of NSCLC patients and have a higher prevalence in patients with adenocarcinoma, women, and those who have never smoked [8, 9, 10]. The frequency of *EGFR* mutation is different among early‐ and late‐stage LUADs [11, 12]. The most common *EGFR* mutations are exon 19 deletion and L858R exon 21 point mutation of the tyrosine kinase domain, accounting for more than 85% of cases [13]. ALK rearrangements have been found in 2–7% of NSCLC patients; they are more common among younger people and nonsmokers [8, 9, 10]. ALK rearrangements are an adverse prognostic factor in surgically resected LUAD patients, while *EGFR* mutations are associated with a better prognosis [10]. Genetic mutations in *KRAS* have been found in 25–35% of NSCLC patients; they occur more often among males, and they are significantly more frequent in smokers or former smokers compared to nonsmokers [4, 8, 9]. The most common mutations in *KRAS* in LUAD are p.G12C, p.G12V, and p.G12D [14]. Proteins encoded by ALK and *EGFR* genes are both tyrosine kinase receptors, while *KRAS*, a small GTPase, is a downstream effector of *EGFR*. They activate the RAF/MEK/MAPK and PI3K/AKT signaling pathways, leading to cell growth and replication [14, 15].

Mass spectrometry (MS)‐based proteomics and phosphoproteomics have a key role in many fields of cancer research, from biomarker discovery and the characterization of disease mechanisms to drug resistance and personalized medicine [16, 17, 18, 19]. Phosphoproteomics is a powerful approach for the characterization of genetic alterations across different cancer types, including LC [20, 21, 22, 23]. Proteomic and phosphoproteomic studies investigating NSCLCs harboring genetic alterations typically focus on identifying dynamic changes upon mutations of specific driver genes, predicting treatment response, and revealing the role of phosphotyrosine signaling in disease pathology [20, 21, 22, 23]. However, the disrupted biological pathways and altered signaling between LUADs with different mutated or translocated driver genes have not been directly/extensively investigated. Therefore, uncovering common and distinct biological processes and phosphorylation events among oncogenic *EGFR*‐mutant, *KRAS* mutant, and *EML4–ALK*‐rearranged LUADs through proteomics and phosphoproteomics analyses could provide significant insights for further studies developing diagnostic approaches or therapeutic strategies for NSCLC.

In this exploratory study, we investigated the altered biological pathways and phosphorylation events primarily on serine and threonine residues in LUAD tumors harboring *EGFR* (*n* = 23), *KRAS* (*n* = 31), or *EML4–ALK* (*n* = 6) oncogenic alterations and triple wild‐type (*n* = 22) LUAD tumors by MS‐based quantitative proteomics and phosphoproteomics analyses of 82 formalin‐fixed paraffin‐embedded (FFPE) tissue sections following on‐surface tryptic digestion of selected tissue regions based on a previously established methodology [24]. The triple wild‐type term refers to tumors without alteration of *EML4–ALK*, *EGFR*, or *KRAS* genes. To the best of our knowledge, this is the first study focusing on altered biological processes and phosphorylation events in LUADs harboring *EML4–ALK*, *EGFR*, or *KRAS* genetic alterations, using small amounts of clinical samples.

## 2 Materials and methods

### 2.1 Materials and tissue samples

All chemicals used were HPLC‐MS grade. Acetonitrile (ACN), water, formic acid (FA), ammonium bicarbonate (ABC), acetic acid, glycerol, phosphorous acid, and trisodium citrate were purchased from Merck (Darmstadt, Germany). Trifluoroacetic acid (TFA), ammonia, dithiothreitol, iodoacetamide, and citric acid were obtained from Thermo Scientific (Unicam, Budapest, Hungary). Methanol, ethanol, and xylene were purchased from VWR International (Debrecen, Hungary). Trypsin Gold and Trypsin/Lys‐C Mix were obtained from Promega (Bio‐Science Hungary, Budapest, Hungary), and RapiGest surfactant was obtained from Waters (Budapest, Hungary). Phospho‐CDK2 (Thr160) antibody (#2561), phospho‐p44/42 MAPK (Erk1/2) (Thr202/Tyr204) (D13.14.4E) XP^®^ Rabbit mAb (#4370) antibody, and phospho‐p38 MAPK (Thr180/Tyr182) (D3F9) XP^®^ Rabbit mAb (#4511) were purchased from Cell Signaling Technology (Kvalitex Kft, Budapest, Hungary).

FFPE lung tissue sections from patients with LUAD were retrospectively collected between January 2008 and December 2020. The study was conducted in accordance with the Declaration of Helsinki. The work was approved by the Medical Research Council of Hungary (TUKEB permit number: IV/2567‐4/2020/EKU). Patient consent was waived with the approval of the Medical Research Council since the study was conducted retrospectively on FFPE‐archived tissue biopsies. FFPE tissue sections were obtained from the departmental archive of the National Korányi Institute of Pulmonology: *EGFR*‐mutated (*n* = 23), *KRAS*‐mutated (*n* = 31), *EML4–ALK* variant (*n* = 6), and triple wild‐type (*n* = 22). All tissue sections were 10 μm thick. Patient material was selected based on the following criteria: surgically resected LUAD specimens from the past several years, and the average age between the groups (63–66) was balanced. Cases included were classified according to the latest WHO histological classification of lung cancer. For the clinical diagnosis of *EGFR* and *KRAS* mutations, tumor regions with the highest tumor cell content were macrodissected from paraffin‐embedded tissue blocks, and DNA was extracted using the MasterPure DNA Purification Kit. *KRAS* mutations were identified through microcapillary‐based restriction fragment length analysis followed by Sanger sequencing, while *EGFR* mutations were detected by polymerase chain reaction (PCR) amplification of exons 18, 19, 20, and 21, followed by bidirectional Sanger sequencing [25]. Additionally, ALK rearrangements were routinely assessed in samples using fluorescence *in situ* hybridization (FISH) [26]. The low number of *EML4–ALK*‐rearranged samples reflects the rarity of this alteration. Summarized information about the samples is presented in Table 1, and detailed information can be found in Table S1.

**Table 1:** Summary of the LUAD samples investigated.

	*EML4–ALK*	*EGFR*	*KRAS*	WT
Number of samples	6	23	31	22
Age	63 (53–72)	66 (39–79)	63 (52–76)	66 (41–76)
Sex				
Female	2	17	19	13
Male	4	6	12	9
Grade				
2	4	16	21	11
2–3	0	1	1	1
3	2	3	7	9
Stage				
I	3	8	8	5
II	2	10	11	7
III	0	5	10	9
IV	1	0	2	0
Smoke				
Never	3	7	0	5
Former	1	6	8	6
Current	0	6	19	10

### 2.2 On‐surface digestion

FFPE tissue sections were handled as previously described [24, 27]. The slides were baked at 60 °C for 2 h to prevent tissue detachment, and then, they were sequentially incubated in solvents with different polarities for deparaffinization: xylene (2 × 5 min), ethanol (2 × 3 min), ethanol–water mixtures (90% ethanol: 3 min, 70% ethanol: 3 min), 10 mm ABC solution (5 min), and water (1 min). Next, heat‐induced antigen retrieval was carried out to disrupt cross‐linking induced by formalin fixation: The tissue sections were boiled at 80–85 °C in citrate buffer (94.6 mm sodium citrate, 20.8 mm citric acid, pH = 6) for 30 min. The digestion was carried out on specific tissue regions characterized by board‐certified pathologists based on the H&E‐stained parallel sections available. In the unstained sections, the selected regions were outlined with a blade to control the digestion area. The proportion of tumor cells relative to other cell types in these regions is presented in Table S1. The exact steps of digestion have been described previously [24]. Briefly, on the tissue surface (ca. 20 mm^2^, using 3–3 μL solutions), the proteins were reduced with RapiGest and dithiothreitol and alkylated with iodoacetamide, and then digested in multiple cycles with LysC‐trypsin mixture (ca. 1 : 25 ratio) and trypsin (ca. 1 : 5 ratio). The extraction of the protein digest was carried out with a 10% acetic acid aqueous solution. The peptide digests from the exact same regions of two parallel tissue sections of the same individual were pooled and treated as one thereafter. Then, the extract was dried and stored at −20 °C until further use.

### 2.3 Purification and phosphopeptide enrichment

Thermo Pierce C_18_ spin columns (Kvalitex) were used for desalting and clean‐up. After each step, the cartridges were centrifuged at 2500 ***g*** for 2 min. The cartridge was conditioned with 2 × 200 μL 50 : 50 *v/v*% methanol : H_2_O and washed with 2 × 200 μL 0.5% TFA in 5 : 95 *v/v*% ACN : H_2_O, then equilibrated with 2 × 200 μL of 0.1% TFA in water. Samples were applied and reapplied once in 50 μL 0.1% TFA in water. The cartridge was washed with 2 × 100 μL of 0.1% TFA, and peptides were eluted with 2 × 50 μL of 0.1% TFA in 30 : 70 *v/v*% H_2_O : ACN. After the purification, the samples were dried down and stored at −20 °C until further use.

For the phosphopeptide enrichment, Pierce TiO_2_ SpinTips (Kvalitex) were used, as previously described [28]. After washing and equilibrating the column, the sample was loaded in 50 mm citric acid buffer and then washed. The flow‐through and the wash fractions were collected for proteomic analysis, and the phosphopeptides were eluted. The proteomic and phosphoproteomic fractions were lyophilized and stored at −20 °C until further use.

The proteomic samples were further cleaned using C_18_ spin columns with the same method described above, while the purification of phosphoproteomic samples was performed on self‐packed (with 10 mg Oasis HLB resin) centrifugal SPE tips [29]. After the elution, the proteomic samples were dried down in the vacuum evaporator, and the phosphoproteomic samples were lyophilized. Both fractions were stored at −20 °C until further use.

### 2.4 Nano‐UHPLC‐MS(MS) analysis—total proteome analysis

Every proteomic sample was reconstituted in 10 μL 0.1% FA in 2 : 98 v/v% ACN : H_2_O, out of which 5 μL was injected. Samples were analyzed using an Orbitrap Exploris™ 240 Mass Spectrometer instrument (Thermo Fisher Scientific, Waltham, MA, USA) equipped with a nanospray ion source coupled to a Waters ACQUITY UPLC M‐Class LC system (Waters, Milford, MA, USA). Peptides were separated on an Acquity M‐Class BEH130 C_18_ analytical column (1.7 μm, 75 μm × 250 mm) using gradient elution (isocratic hold at 3% for 1 min, then elevating B solvent content to 25% in 81 min, and to 40% in 3 min) following 3 min trapping on an M‐Class Symmetry C_18_ (100 Å, 5 μm, 180 μm × 20 mm) trap column. Solvent A consisted of 0.1% FA in water, and solvent B was 0.1% FA in ACN.

For MS analysis, data‐dependent analysis (DDA) measurements were performed. The number of dependent scans was set at 20, with a dynamic MS/MS exclusion of the same precursor ion for 40 s if it occurred more than 2 times within 30 s. Preferred charge states were set between +2 and +6. MS spectra were acquired in the 360–2200 *m/z* range, while the scan range was selected automatically for the MS/MS spectra.

### 2.5 Nano‐UHPLC‐MS(MS) analysis—phosphoproteomic analysis

Every phosphoproteomic sample was reconstituted in 8 μL 0.1% FA in 2 : 98 v/v% ACN : H_2_O, out of which 6 μL was injected. The samples were analyzed on a nanoElute 2 nano‐HPLC coupled to a timsTOF HT (Bruker Daltonics, Billerica, MA, USA) equipped with a CaptiveSpray 1 source. Phosphopeptides were separated on an experimental monolithic C_18_ column (150 mm*30 μm ID, 0.46 μL void volume, Bruker Daltonics) heated at 50 °C using gradient elution (constant 0.50 μL·min^−1^ flow; elevating B solvent content from 5% to 17% in 7 min, and 35% in 3 min, followed immediately by a 5 min plateau at 95%). Trapping was performed on an Acclaim PepMap trap column (5 μm C_18_‐coated particles, 300 μm × 5 mm, ThermoFisher Scientific P/N 160454). Solvent A consisted of 0.1% FA in water, and solvent B was 0.1% FA in ACN.

MS settings were the following: *m/z* range = 100–1700 Th, ion mobility range = 0.84–1.3 1/K0; transfer time = 60 μs, pre‐pulse storage time = 12 μs, ion polarity = Positive, scan mode = MS/MS (Pasef); TIMS parameters: ramp time = 180 ms, accumulation time = 180 ms; PASEF parameters: ms/ms scans = 4, total cycle time = 0.93068 s, charge range = 0–5, intensity threshold for scheduling = 1500, scheduling target intensity = 15 000, exclusion release time = 0.4 min, reconsider precursor switch = on, current/previous intensity ratio = 4, exclusion window mass width = 0.015 m/z, exclusion window v·s·cm^−2^ width = 0.015 V·s·cm^−2^.

### 2.6 Immunohistochemistry

Formalin‐fixed and paraffin‐embedded human lung tissue sections (2 μm thick) were deparaffinized and rehydrated. Heat‐induced antigen retrieval was performed using BOND Epitope Retrieval Solution 2 (AR9640, Leica Biosystems, Newcastle, UK). Endogenous peroxidase blocking was carried out using 10% H_2_O_2_ in methanol for 20 min at room temperature. Sections were washed in phosphate‐buffered saline containing 0.1% v/v Tween‐20 (PBST; containing 137 mm of NaCl, 2.7 mm of KCl, 10 mm of Na_2_HPO_4_, and 1.8 mm of KH_2_PO_4_, pH 7.5) followed by blocking the non‐specific protein binding sites with 10% bovine serum albumin in PBST (BSA, A6003, Merck KGaA, Darmstadt, Germany) for 1 h at room temperature. The sections were incubated with phospho‐p44/42 MAPK (1 : 1000), phospho‐p38 MAPK (1 : 500), and phospho‐CDK2 (1 : 100) antibodies or with the antibody diluent for the no primary antibody controls overnight at 4 °C in 1 : 2000 concentration. The negative control staining was prepared by omitting the primary antibody. The sections were washed and incubated with the HISTO‐Labeling System for 40 min at room temperature (30011.R500, Department of Immunology and Biotechnology, Pécs, Hungary). Diaminobenzidine 3 : 100 (ImmPACT DAB, SK‐4105, Vector Laboratories, Burlingame, CA, USA) was used to visualize the reaction, followed by counterstaining with hematoxylin. The sections were covered using BIOMOUNT (BMT‐500, BIOGNOST D.O.O, Zagreb, Croatia) and scanned with the Pannoramic P1000 scanner. Isotype controls were used as negative controls.

### 2.7 Data analysis

Software used: byonic 5.0.20 [30] (https://proteinmetrics.com, accessed on 6 June 2024), maxquant 2.4.0.0 and 2.4.2.0 [31] (https://maxquant.org, accessed on 6 June 2024), r 4.3.2 [32] (https://www.r‐project.org/, accessed on 6 June 2024), rstudio 2023.06.1 [33] (https://posit.co/, accessed on 6 June 2024), string 12.0 [34] (https://string‐db.org/, accessed on 6 June 2024), and ksea app 1.0 [35, 36] (https://casecpb.shinyapps.io/ksea/, accessed on 6 June 2024). The exact parameters used for all the software are summarized in Table S2.

Protein identification was performed by byonic on the *Homo sapiens* database (82 427 sequences, downloaded from https://www.uniprot.org/ in July 2023). Protein quantitation on the proteomic dataset was performed using maxquant v2.4.0.0 on a focused database, made from merging byonic search results from all proteomic MS/MS analyses. The number of quantified proteins varied between 1377 and 2189 across the samples. After quantitation, the data were log2 transformed and filtered based on the number of missing values in each of the four sample groups: only proteins found in at least 50% of all samples in at least one sample group (*KRAS*, *EGFR*, WT) were kept for further analysis. Regarding the *EML4–ALK* sample group, proteins had to be found in four out of six samples. Missing values were then imputed in a group‐wise manner according to the following: If the protein in question was found in less than 1/2 of all samples in the group, it was imputed with the sample 5‐percentile, while if it was found in at least 1/2 of all samples in the group, it was imputed according to the kNN algorithm (vim package [37], *k* = 10, similarity based on Euclidean distances).

Phosphosite quantitation on the phosphoproteomic dataset was performed using maxquant v2.4.2.0 on a focused database made from merging byonic search results from all phosphoproteomic MS/MS analyses. The number of quantified phosphosites varied between 73 and 1781 across the samples. After quantitation and log2 transformation, phosphosites were filtered based on the localization probability (≥ 0.75) and the number of missing values similar to the proteomic dataset. The missing values were then imputed using the site‐ and condition‐specific imputation if the quantitation rate within that condition was ≥ 0.5 (scImpute function in phosr package [38]). The remaining sites were imputed using tail‐based imputation (tImpute function in phosr package). The imputed dataset was normalized for sample medians using medianScaling function (scale = *F*, phosr package).

The following statistical analysis was carried out on both datasets: Normality was tested using Shapiro–Wilk tests within each sample group, and variances between the groups compared were tested using Levene tests. For multiple group comparisons, analysis of variance (ANOVA), Welch‐ANOVA, and Kruskal–Wallis tests were performed based on the outcome of the normality and equality of variances tests. For two‐group comparisons on proteins/phosphosites significant in the multiple group comparison, Student's *t*‐tests, Welch *t*‐tests, and Wilcoxon rank sum tests were performed based on the outcome of the normality and equality of variances tests. False discovery rates were controlled separately for every protein/phosphosite using the Benjamini–Hochberg method at 5%. Plots were made using the ggplot2 [39] and ggpattern packages. The uniform manifold approximation and projection (UMAP) was performed using the umap function from the m3c package [40], and hierarchical clustering was performed using the heatmap.3 function [41] (using Ward's clustering method ‘ward.D2’ from the hclust function). For the UpSet plot, plot_upset function was used from msnset.utils package [42].

Gene set enrichment analysis (GSEA) was performed using the gseGO function in the clusterprofiler r package [43] for Gene Ontology Biological Process (GOBP) terms. The genes were preranked based on effect sizes (Cohen's *d*). Enriched terms were visualized using the dotplot and the gseaplot2 functions in the clusterprofiler package.

Functional enrichment of phosphoproteins was performed in string for Gene Ontology (GO) terms. The minimum required interaction score was set to medium confidence (0.400). For the visualization, the following parameters were used: Number of terms shown was set to 10, and group terms by similarity ≥ 0.8.

Kinases were predicted for the 183 substrates significantly dysregulated. Substrate scores for kinases were generated using the PhosphoSitePlus [44] database and the ‘kinaseSubstrateScore’ function in phosr package based on the features of phosphorylation profiles and sequence motifs. Kinase was predicted for a given substrate using the ‘kinaseSubstratePred’ function in phosr package, a machine‐learning approach utilizing adaptive sampling. Kinase network was constructed using ‘plotKinaseNetwork’ function in phosr package.

Relative kinase activities were inferred using KSEA App. The analyses were performed separately for the six comparisons. PhosphoSitePlus and networKIN [45] were the selected kinase–substrate datasets.

## 3 Results

**Fig. 1:**
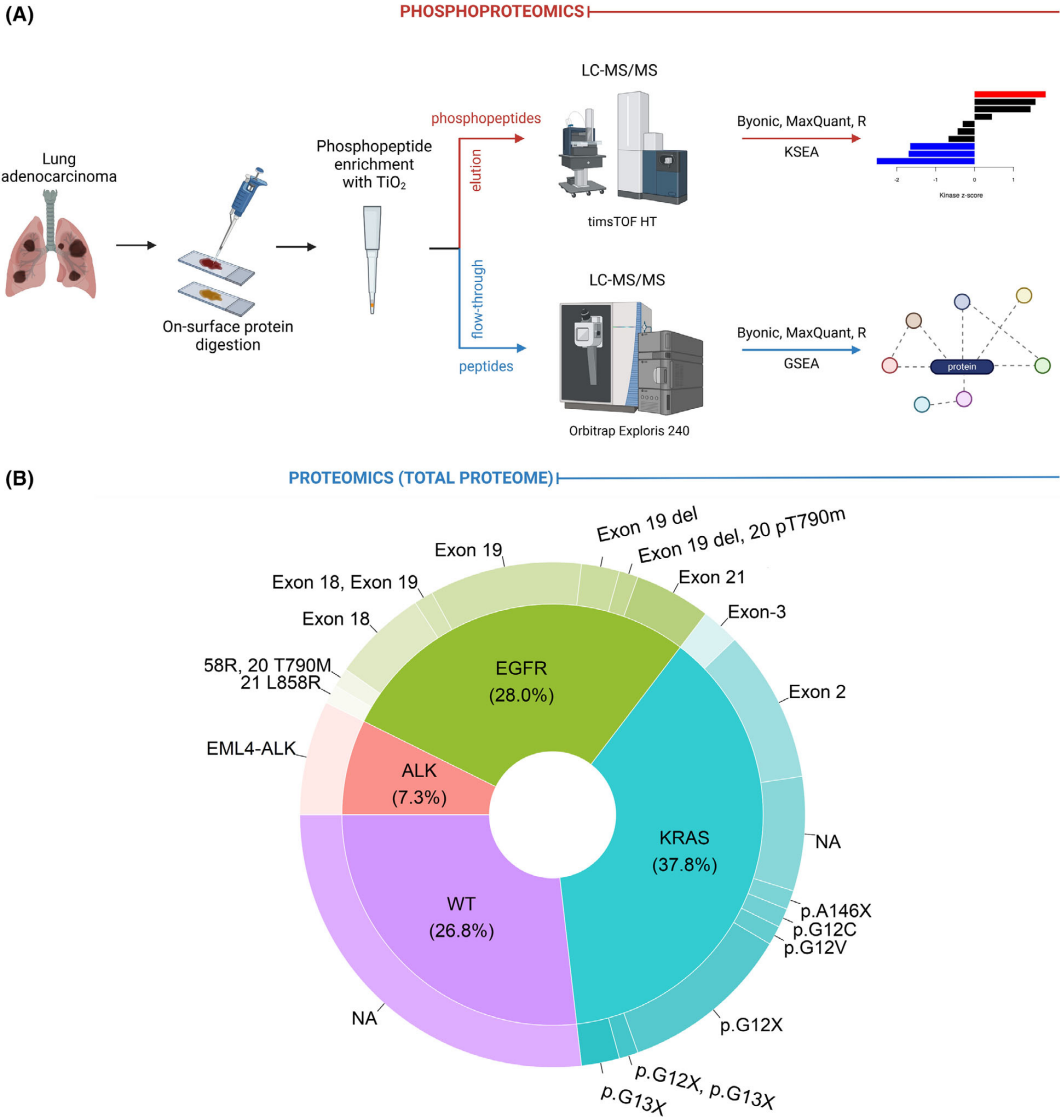
(Phospho)proteomic analysis of lung adenocarcinoma (LUAD) tumors. (A) Schematic workflow of applied methodologies: Small regions of formalin‐fixed paraffin‐embedded (FFPE) tissue sections from LUAD tumor samples were selected and subjected to on‐surface tryptic digestion. The resulting peptides were enriched for phosphopeptides using TiO_2_ spin tips, separating phosphopeptides from non‐phosphorylated peptides, both of which were then analyzed separately by mass spectrometry. (B) Distributions of the samples analyzed in the cohort based on the genetic alterations and subtypes. In the case of WT samples, NA refers to not applicable, while for KRAS samples, NA refers to not available.

Proteomic and phosphoproteomic analyses were performed following on‐surface digestion of small regions of FFPE tissue sections from individuals diagnosed with LUAD. The tryptic digest of each sample extracted from the tissue surface of defined regions of two parallel sections was combined and subjected to phosphopeptide enrichment using TiO_2_ spin tips. Phosphopeptides containing phosphorylation on serine or threonine residues were predominantly enriched. The phosphopeptides were then separated from the non‐phosphorylated peptides, and the two fractions were analyzed in separate mass spectrometry runs (Fig. 1A). The samples were grouped into four sample groups based on the type and presence of a given genetic alteration (*EML4–ALK*‐rearranged, *EGFR*‐mutated, *KRAS*‐mutated, and triple wild‐type), and statistical analysis was performed accordingly. The sample groups contained 6, 23, 31, and 22 samples, respectively (Fig. 1B). The low number of *EML4–ALK*‐rearranged samples represents the frequency of the alteration.

Patient and sample information are summarized in Table 1; detailed information regarding the samples is available in Table S1. The results of the proteomic and phosphoproteomic analyses are discussed separately.

### 3.1 Proteomics—identification of proteins and biological processes specific to samples with different genetic alterations through total proteomics

During the total proteomic analysis, 3672 proteins were quantified by maxquant using label‐free quantitation. The number of quantified proteins per sample ranged from 1377 to 2189. Following initial filtering based on the number of missing values, 2021 proteins were subjected to statistical analysis. The methods and parameters used for data analysis are summarized in Table S2.

**Fig. 2:**
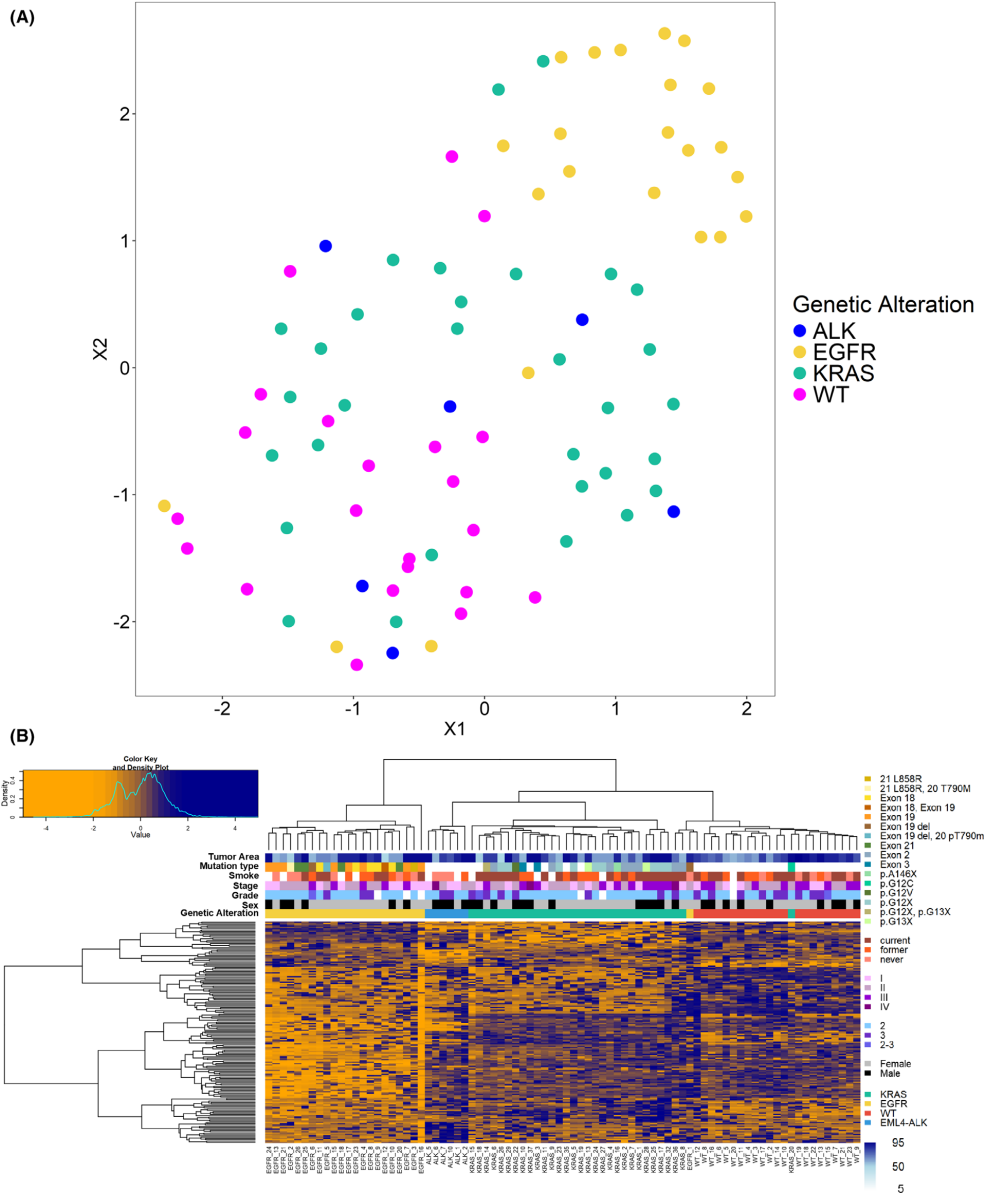
Proteomic analysis of the *EML4–ALK*, *EGFR*, *KRAS*, and triple wild‐type (WT) sample groups. (A) Uniform manifold approximation and projection (UMAP) representation of the 82 samples based on the 2021 proteins. Different colors mark the four sample groups (blue for *EML4–ALK*, yellow for *EGFR*, green for *KRAS*, and magenta for wild‐type). (B) Heatmap with hierarchical clustering of the 166 proteins significantly altered (false discovery rate < 0.05, fold change > 2) between the groups (log2 protein LFQ intensity values are *Z*‐scored).

Uniform manifold approximation and projection (UMAP) was performed based on the 2021 proteins. Most of the *EGFR*, *KRAS*, and WT samples clustered separately, while *EML4–ALK*‐rearranged samples grouped together with the other subtypes (Fig. 2A). This could reflect the molecular heterogeneity within the *EML4–ALK*‐rearranged group, where variations in protein expression might influence clustering behavior. Proteomic data were further investigated to identify proteins differentially expressed between the sample groups. Altogether, in at least one of the six two‐group comparisons (*EML4–ALK*‐*EGFR*, *EML4–ALK*‐*KRAS*, *EML4–ALK*‐WT, *EGFR*‐*KRAS*, *EGFR*‐WT, *KRAS*‐WT), the expression of 504 proteins was significantly altered (FDR < 0.05), out of which 166 proteins had a fold‐change of over 2. The results of the two‐group comparison tests (test type, *P*‐value, fold‐change) for the 504 proteins are summarized in Table S3. Hierarchical clustering based on the 166 proteins revealed distinct molecular profiles among samples with different genetic alterations (Fig. 2B). Moreover, *EGFR*‐mutated samples clustered separately from the other three groups. However, it is important to note that the clustering was not influenced by other clinical parameters such as tumor area, mutation subtype, smoking history, stage, grade, or sex. Furthermore, samples with 5–10% tumor content, including those from *EGFR*_12, *EGFR*_2, *KRAS*_18, and *KRAS*_26, clustered together with other samples harboring the same mutation.

Several proteins showed exceptionally high fold‐changes (above 8‐fold); for example, 15‐hydroxyprostaglandin dehydrogenase (PGDH) was significantly overexpressed, while cingulin was significantly under‐expressed in *EML4–ALK* compared to the other three sample groups. Cysteine‐rich protein 1 (CRIP1) and protein S100‐P were significantly overexpressed in *KRAS* and WT groups compared to *EML4–ALK* and *EGFR* groups.

Multiple proteins related to *EGFR*, *EML4–ALK*, or *KRAS* signaling pathways showed significant differences in their expression between the sample groups (FDR < 0.05). Receptor tyrosine protein kinase erbB‐2 (ERBB2) [46] was significantly altered in *EGFR* and *KRAS* groups compared to the WT group; SHC‐transforming protein 1 (SHC1) [47] was significantly altered in *EML4–ALK* and *KRAS* groups compared to the *EGFR* group. Additional proteins that are indirectly regulated by these pathways were also significantly altered between the groups; for example, caspase‐7 [48] was under‐expressed in *EML4–ALK*, and metalloproteinase‐9 [49] in *KRAS* compared to the other sample groups.

**Fig. 3:**
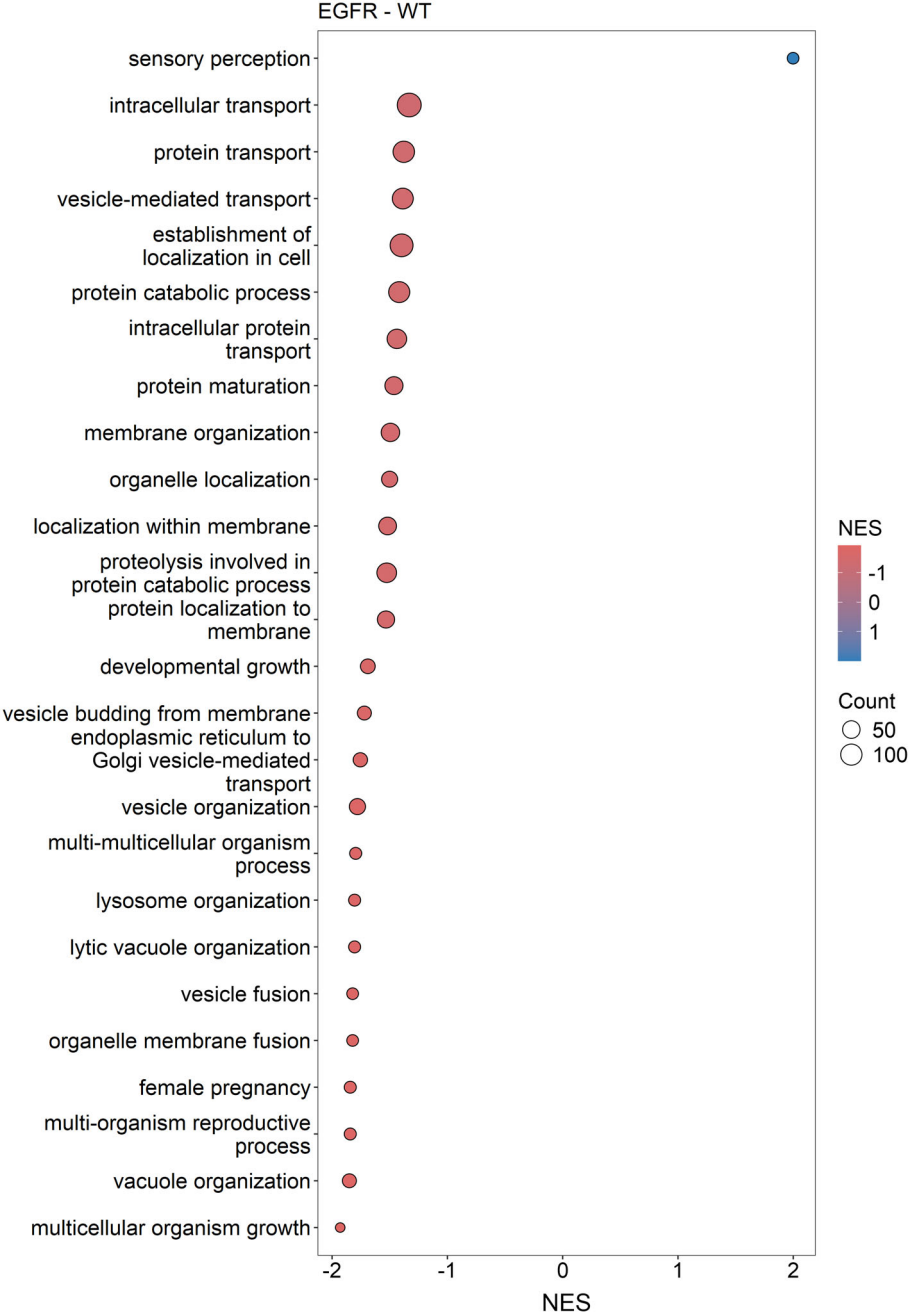
Gene set enrichment analysis (GSEA) dot plot of significantly enriched Gene Ontology Biological Process (GOBP) terms in *EGFR*‐WT comparison, where the x‐axis represents the normalized enrichment score (NES). The size of the dots corresponds to the number of genes associated with the term, and the color represents the NES.

Gene set enrichment analysis was performed in R for Gene Ontology (GO) Biological Process (BP) terms, based on the 2021 proteins. The complete lists of enriched terms are provided in Table S4. Most of the enriched terms were related to vesicle organization, transport, homeostasis, metabolic, and translation biological processes. Of the 79 enriched GOBP terms, only a few showed potential genetic alteration specificity. For example, membrane organization, vesicle organization, and vesicle‐mediated transport terms were significantly suppressed in *EGFR*‐mutated samples compared to the other sample groups. A dot plot for the enriched terms in the *EGFR*‐WT comparison is presented in Fig. 3. Dot plots for the other comparisons are provided in Figs S1–S5, and GSEA plots of significantly enriched GOBP terms in the comparisons of the *EGFR* sample group are presented in Figs S6–S8.

**Fig. 4:**
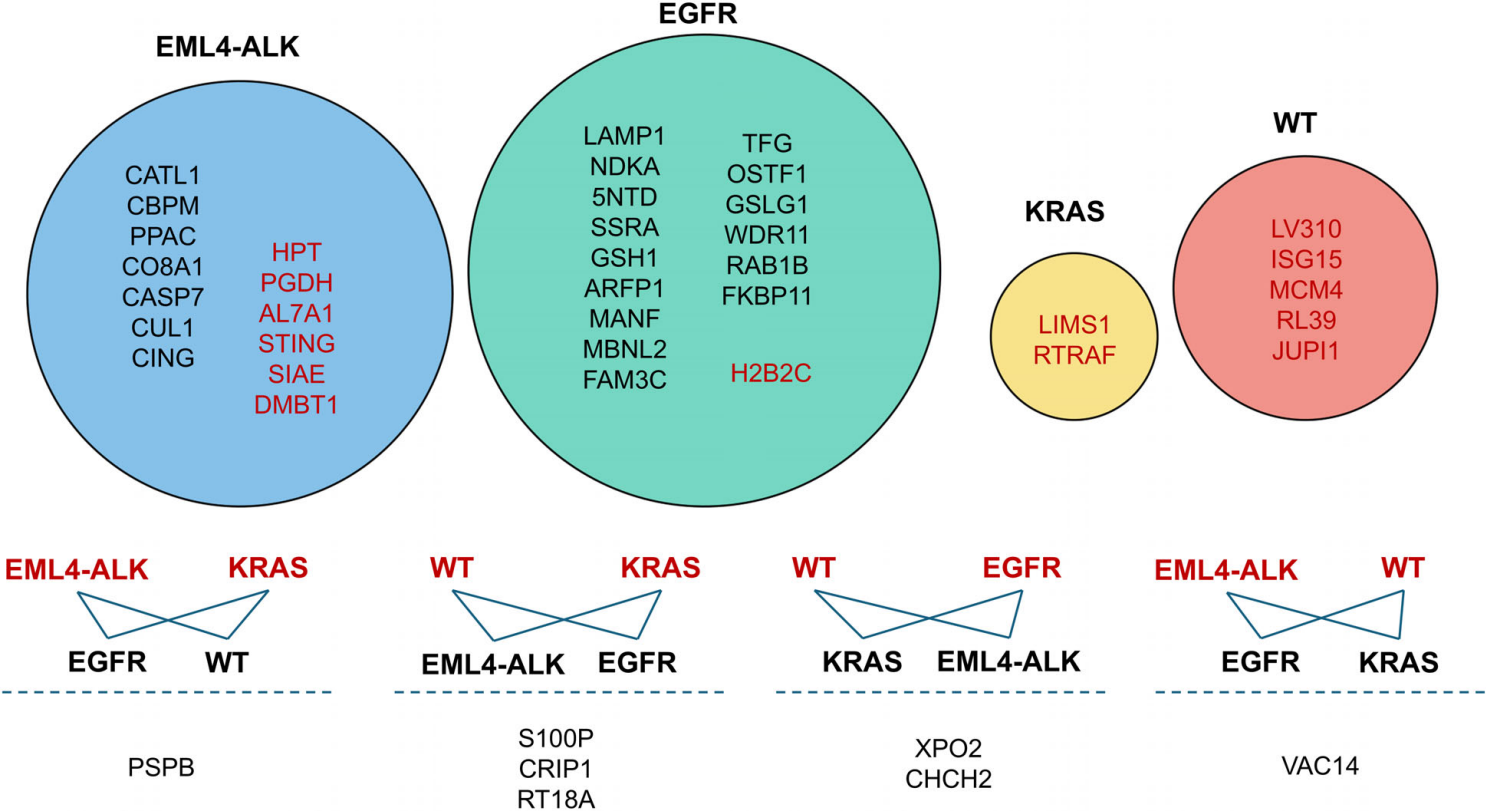
Euler plot of the 36 potential alteration‐specific proteins. Proteins highlighted in black were significantly under‐expressed, while those in red were significantly overexpressed within the specific sample group. The proteins positioned at the bottom of the figure exhibit distinct expression patterns across multiple groups. A blue line indicates significant differences in expression between the connected groups, with overexpression occurring in the sample groups highlighted in red.

Altogether, 36 proteins were found to be differentially expressed (FDR < 0.05 and fold‐change above 2) in all three comparisons of the sample groups, identifying them as potentially genetic alteration‐specific (Fig. 4). For example, procathepsin L (CATL1) was significantly under‐expressed in *EML4–ALK*‐rearranged samples compared to *EGFR*‐mutated, *KRAS*‐mutated, and WT samples. Besides the 16, 13, 2, and 5 proteins altered specifically in *EGFR*, *EML4–ALK*, *KRAS*, and WT groups, an additional seven proteins exhibited distinct expression differences across multiple groups. For example, pulmonary surfactant‐associated protein B (PSPB) was found to be significantly overexpressed in *EML4–ALK* and *KRAS* groups compared to *EGFR* and WT groups (Fig. 4).

### 3.2 Phosphoproteomics—identification of unique phosphosites and inference of kinases specific to samples with different genetic alterations

Altogether, 3708 phosphosites were quantified by maxquant, and the number of quantified phosphosites per sample ranged from 73 to 1781. Out of the 3708 phosphosites, 384 sites were selected for further statistical analysis based on the number of missing values. These 384 phosphosites were derived from 254 unique phosphorylated proteins. The methods and parameters used for data analysis and statistical analysis are summarized in Table S2.

**Fig. 5:**
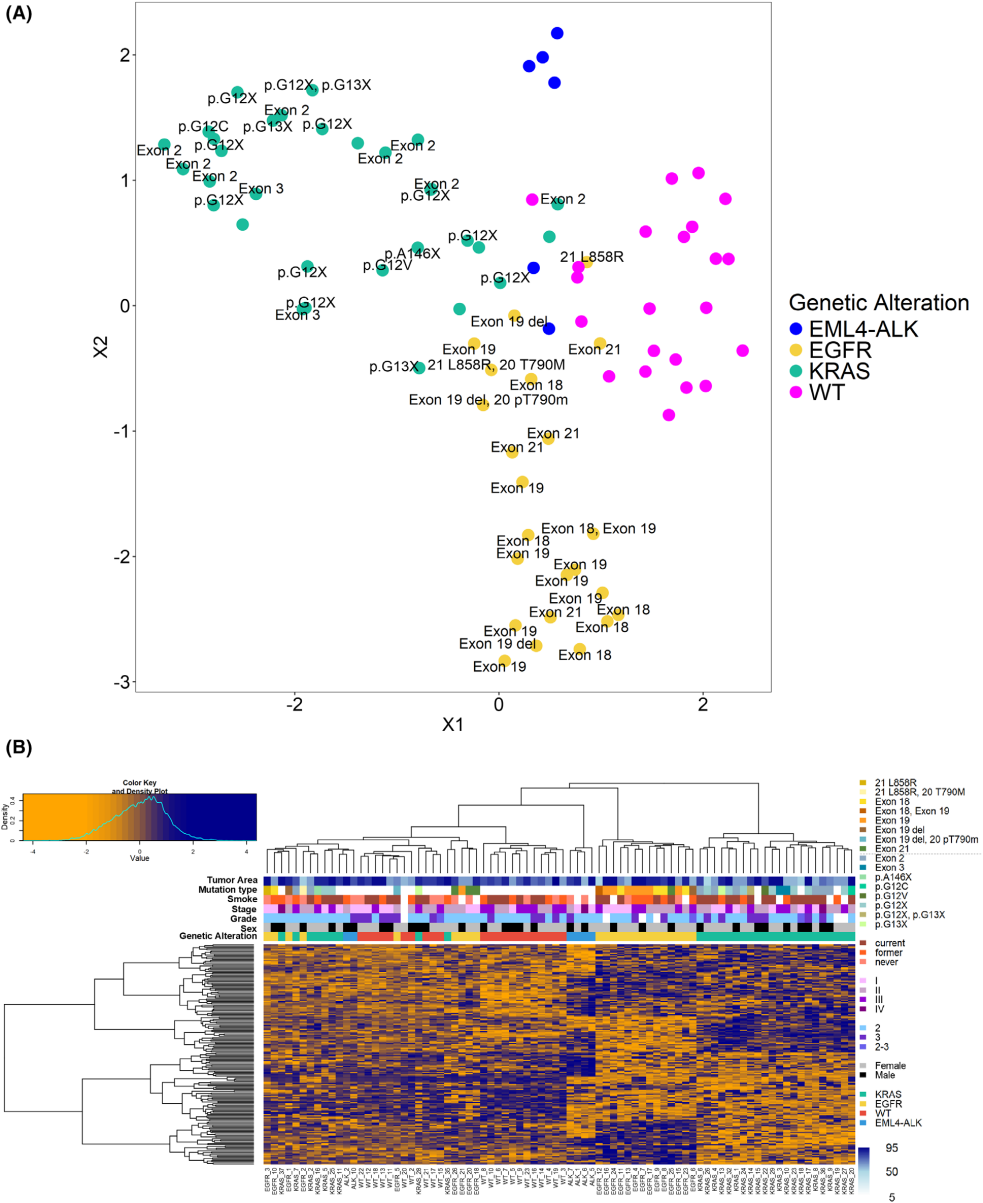
Phosphoproteomic analysis of the *EML4–ALK*, *EGFR*, *KRAS*, and WT sample groups. (A) UMAP representation of the 82 samples based on the 384 phosphosites quantified using maxquant and considered for statistical analysis. Different colors mark the four sample groups (blue for *EML4–ALK*, yellow for *EGFR*, green for *KRAS*, and magenta for wild‐type). (B) Heatmap with hierarchical clustering of the 183 phosphosites significantly altered (false discovery rate < 0.05, fold change > 2) between the groups (log2 phosphosite intensity values are *Z*‐scored).

Dimensionality reduction was performed using UMAP based on the 384 phosphosites (Fig. 5A). Most of the *EML4–ALK*, *EGFR*, *KRAS*, and WT samples formed separate clusters. Moreover, most of the samples with *EGFR* Exon 18 and Exon 19 mutations clustered distinctly from those with *EGFR* Exon 21 mutations. Phosphoproteomic data were further investigated to identify phosphosites differentially phosphorylated between the four sample groups. Altogether, in at least one of the six two‐group comparisons, the phosphorylation of 211 sites was significantly altered (FDR < 0.05), out of which 183 had a fold‐change over 2. The results of the two‐group comparison tests (test type, *P*‐value, fold‐change) for the 211 phosphosites are summarized in Table S5. Hierarchical clustering based on the 183 phosphosites revealed some interesting differences between the samples with different genetic alterations (Fig. 5B). Most of the *EML4–ALK* and *KRAS* samples formed a distinct cluster, separate from the other samples, while most of the *EGFR* samples clustered separately from the WT and the other samples. However, samples with *EGFR* Exon 21 mutation clustered together with other genetic alterations or WT samples. Approximately one‐third of the samples clustered together regardless of their genetic alteration. This is because the highest numbers of proteins and phosphosites were identified in these samples. The cases with 5–10% tumor content (EGFR_12, KRAS_18, and KRAS_26) did not exhibit any distinct clustering behavior in the analyses, as they were part of the broader clusters formed by their respective genetic alterations. These samples did not have fewer identified phosphosites compared to those with higher tumor content, and the overall clustering was influenced more by the mutation status and the number of identified components rather than tumor content. Notably, one *EGFR* sample, EGFR_2 (with 10% tumor content), did not cluster with the other *EGFR* samples but rather with samples that had a higher number of identified phosphosites. Similarly, two *EML4–ALK* samples (with 816 and 1055 identified phosphosites) clustered apart from the others, which had lower phosphosite numbers (86, 87, 120, and 236).

Note that two phosphorylation sites in EGFR (S946 and T648) have been quantified in the phosphoproteomic dataset; however, due to the huge number of missing values, they had been filtered out during the initial filtering. Additionally, the EGFR protein has also been quantified in the proteomic dataset. However, the protein itself did not show significant differences during the statistical tests.

To assess whether the phosphorylation or the protein expression was altered, we compared the fold‐change of each significant phosphosite to the fold‐change of the corresponding protein determined from the proteomic dataset separately for all six comparisons (Figs S9–S14). We observed a phosphorylation site (KRR1;S3) in the *EGFR*‐*KRAS* comparison where the change in phosphorylation was 2.0‐fold while the corresponding protein change was 1.7‐fold. Additionally, for the PML;S518 site in the *EML4–ALK*‐*EGFR* comparison, both the site and the protein exhibited approximately a 2‐fold increase, suggesting that in these cases, the change in phosphorylation was likely driven by an increase in protein abundance. However, in all the other cases, changes in protein abundance could be excluded as the primary cause of the change in the level of phosphorylation.

To uncover upstream kinases responsible for the changes in phosphorylation of the significantly altered phosphosites, potential kinase–substrate pairs and global relationships between kinases were identified using the phosr package (Figs S15 and S16). The combined score of substrates and the predicted kinase–substrate scores are presented in Table S6.

**Fig. 6:**
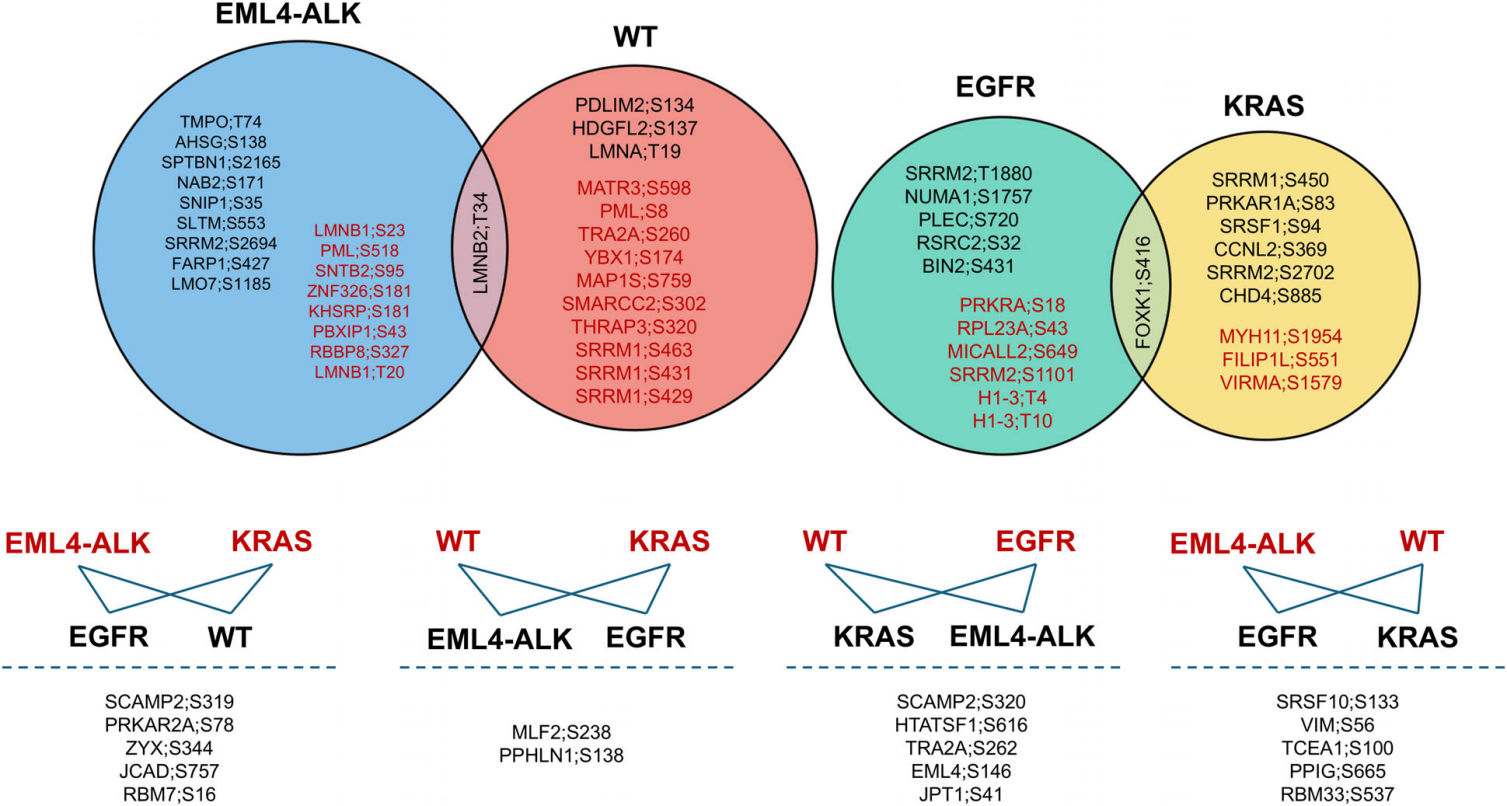
Euler plot of the 52 potential alteration‐specific phosphosites. Phosphosites highlighted in black showed significantly decreased phosphorylation, while those in red showed significantly increased phosphorylation within the specific sample group. The phosphosites positioned at the bottom of the figure exhibit distinct phosphorylation patterns across multiple groups. A blue line indicates significant differences in phosphorylation between the connected groups, with increased phosphorylation occurring in the sample groups highlighted in red.

Out of the 183 phosphosites significantly altered in at least one of the six comparisons, 52 sites were specifically differentially phosphorylated in one sample group (Table 2). Altogether, 18, 12, 10, and 14 phosphosites were altered in all three comparisons of the *EML4–ALK*, *EGFR*, *KRAS*, and WT sample groups (Fig. 6). Additionally, 17 phosphosites showed distinct phosphorylation differences across multiple groups. For example, a higher level of phosphorylation of secretory carrier‐associated membrane protein 2 (SCAMP2) at S319 was found in *EML4–ALK* and *KRAS* groups compared to *EGFR* and WT groups.

**Table 2:** List of potentially alteration‐specific phosphosites (significantly dysregulated in all three comparisons of a specific genetic alteration or the WT samples based on the phosphoproteomic analysis) supplemented with information from PhosphoSitePlus.

Site	Alteration specificity	Direction of change	Downstream effects on biological processes
TMPO;T74	*EML4–ALK*	DOWN	–
AHSG;S138	*EML4–ALK*	DOWN	Carcinogenesis, induced
SPTBN1;S2165	*EML4–ALK*	DOWN	–
NAB2;S171	*EML4–ALK*	DOWN	–
SNIP1;S35	*EML4–ALK*	DOWN	–
SLTM;S553	*EML4–ALK*	DOWN	–
SRRM2;S2694	*EML4–ALK*	DOWN	–
FARP1;S427	*EML4–ALK*	DOWN	–
LMO7;S1185	*EML4–ALK*	DOWN	–
LMNB1;S23	*EML4–ALK*	UP	–
PML;S518	*EML4–ALK*	UP	Apoptosis, inhibited; carcinogenesis, induced and inhibited; cell growth, induced; cell motility, induced; signaling pathway regulation; transcription, induced
SNTB2;S95	*EML4–ALK*	UP	–
ZNF326;S181	*EML4–ALK*	UP	–
KHSRP;S181	*EML4–ALK*	UP	–
PBXIP1;S43	*EML4–ALK*	UP	–
RBBP8;S327	*EML4–ALK*	UP	Cell cycle regulation
LMNB1;T20	*EML4–ALK*	UP	–
LMNB2;T34	*EML4–ALK*, WT	*EML4–ALK* > *EGFR*, *KRAS* > WT	–
SRRM2;T1880	*EGFR*	DOWN	–
NUMA1;S1757	*EGFR*	DOWN	–
PRKRA;S18	*EGFR*	UP	–
RPL23A;S43	*EGFR*	UP	–
MICALL2;S649	*EGFR*	UP	–
SRRM2;S1101	*EGFR*	UP	–
H1‐3;T4	*EGFR*	UP	–
H1‐3;T10	*EGFR*	UP	–
PLEC;S720	*EGFR*	DOWN	–
RSRC2;S32	*EGFR*	DOWN	–
BIN2;S431	*EGFR*	DOWN	–
FOXK1;S416	*EGFR*, *KRAS*	*EGFR* > *EML4–ALK*, WT > *KRAS*	–
SRRM1;S450	*KRAS*	DOWN	–
MYH11;S1954	*KRAS*	UP	–
FILIP1L;S551	*KRAS*	UP	–
VIRMA;S1579	*KRAS*	UP	–
PRKAR1A;S83	*KRAS*	DOWN	Carcinogenesis, induced; cell cycle regulation
SRSF1;S94	*KRAS*	DOWN	–
CCNL2;S369	*KRAS*	DOWN	–
SRRM2;S2702	*KRAS*	DOWN	–
CHD4;S885	*KRAS*	DOWN	–
MATR3;S598	WT	UP	–
PDLIM2;S134	WT	DOWN	–
PML;S8	WT	UP	Apoptosis, altered
TRA2A;S260	WT	UP	–
YBX1;S174	WT	UP	Transcription, altered
MAP1S;S759	WT	UP	–
SMARCC2;S302	WT	UP	–
THRAP3;S320	WT	UP	–
SRRM1;S463	WT	UP	–
SRRM1;S431	WT	UP	–
SRRM1;S429	WT	UP	–
HDGFL2;S137	WT	DOWN	–
LMNA;T19	WT	DOWN	Cell cycle regulation

The phosphosites potentially specific to a given genetic alteration were derived from 44 phosphorylated proteins. None of these phosphoproteins showed significant alteration at the proteome level (FDR < 0.05 and fold‐change above 2). Serine/arginine repetitive matrix protein 1 (SRRM1), SRRM2, lamin‐B1, protein PML, and histone H1.3 had multiple phosphosites significantly altered in at least one sample group. Interestingly, the phosphorylation of SRRM2 at T1880 was found to be decreased in *EGFR*, while phosphorylation at S1101 was increased in *EGFR*, and phosphorylation at S2702 was decreased in *KRAS* compared to the other three sample groups.

**Fig. 7:**
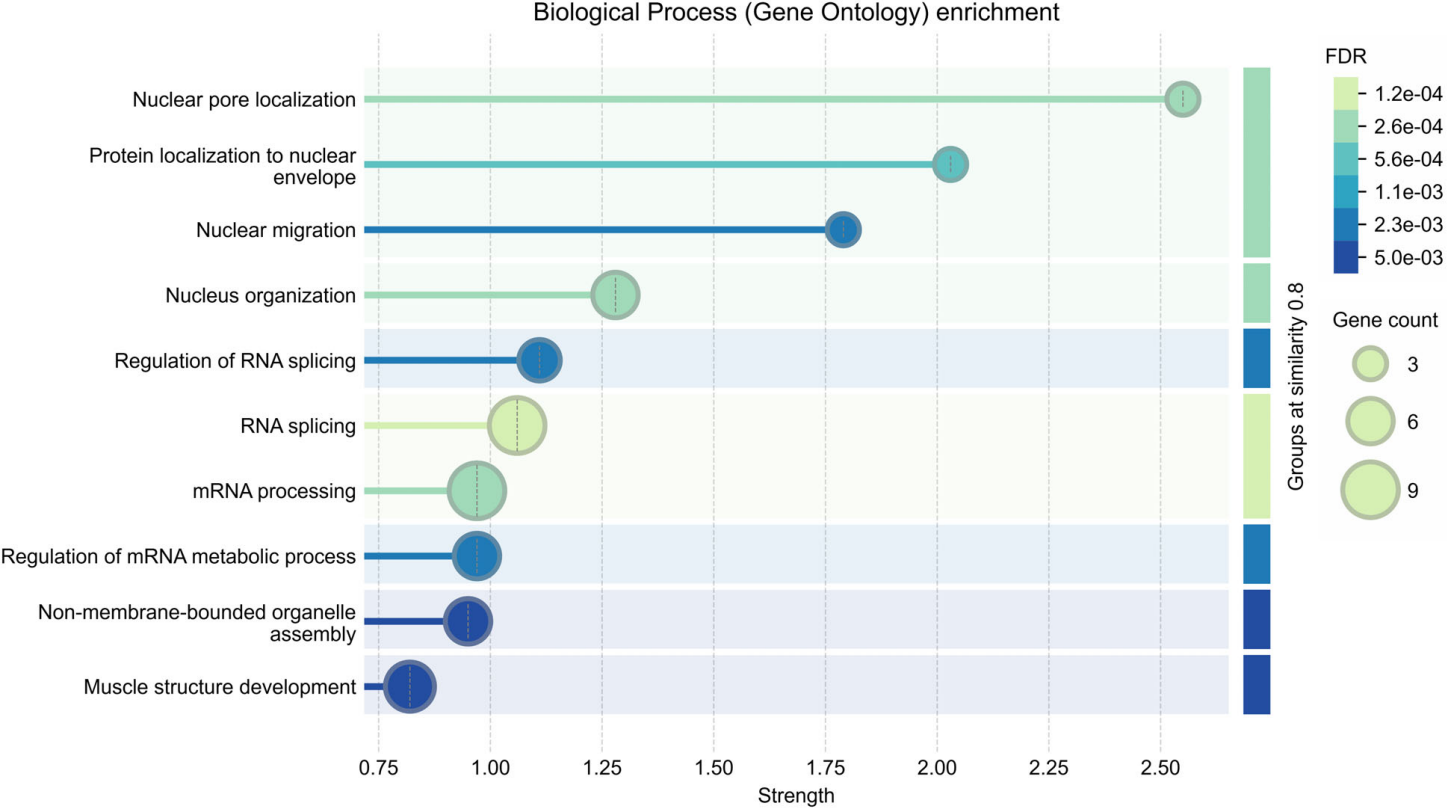
Visualization of functional enrichment analysis in string for Gene Ontology Biological Process (GOBP) terms of 44 phosphoproteins with potential alteration‐specific phosphosites. The size of the dots corresponds to the number of genes associated with the term, and the color represents the false discovery rate (FDR).

A protein–protein interaction (PPI) network of the 44 phosphoproteins was constructed in STRING for GO terms. Functional enrichment analysis revealed that many of these proteins were most strongly associated with the following GOBP terms: cellular localization, nuclear migration, nucleus organization, regulation of RNA splicing, and RNA splicing (Fig. 7). The complete lists of enriched terms are presented in Table S7; the visualization of Gene Ontology Molecular Function (GOMF) and Gene Ontology Cellular Component (GOCC) terms is presented in Figs S17 and S18.

### 3.3 Kinase–substrate enrichment analysis

**Fig. 8:**
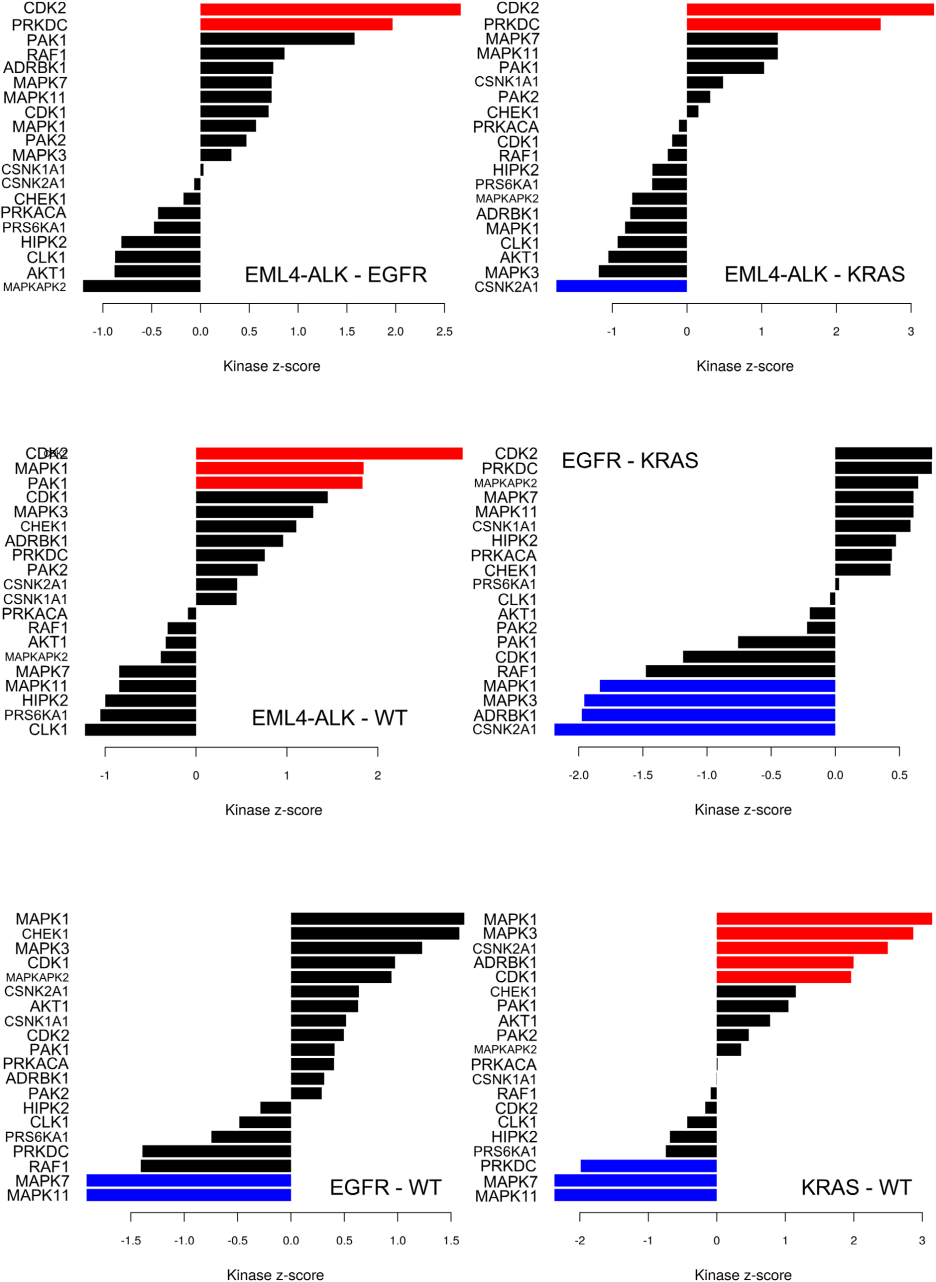
Result of the kinase–substrate enrichment analysis (KSEA). Kinase scores based on KSEA of 202 kinase–substrate pairs (number of substrates ≥ 3). Blue bars for suppressed and red bars for elevated kinase activities (*P* < 0.05 based on z‐score transformation). Black bars for kinases without significantly altered activities.

Kinase activities were inferred based on substrate phosphorylation levels by kinase–substrate enrichment analysis (KSEA) [36]. The activity of a given kinase is deduced by comparing the quantitative values of the identified substrates of the given kinase and the quantitative values of all substrates identified in the sample. Two‐group comparisons were performed between the four sample groups; altogether 202 kinase–substrate pairs were analyzed in the case of all six comparisons. KSEA revealed 81 kinases with at least one identified substrate (Table S8). Cyclin‐dependent kinase 1 (CDK1) had the largest number of substrates (*m*), 19. Altogether, 10 kinases showed significantly altered activities (*m* ≥ 3, *P* < 0.05) in at least one of the six comparisons (Fig. 8). The activity of cyclin‐dependent kinase 2 (CDK2) was significantly elevated in *EML4–ALK* samples compared to all other sample groups. The activity of casein kinase 2 alpha 1 (CSNK2A1) was significantly elevated in *KRAS* compared to the other groups. Additionally, the activity of mitogen‐activated protein kinase 1 (MAPK1) was elevated in both *EML4–ALK* and *KRAS*‐mutated samples compared to the WT, while the activities of mitogen‐activated protein kinase 7 (MAPK7) and mitogen‐activated protein kinase 11 (MAPK11) were suppressed in both the *EGFR*‐mutated and *KRAS*‐mutated samples relative to the WT samples.

**Fig. 9:**
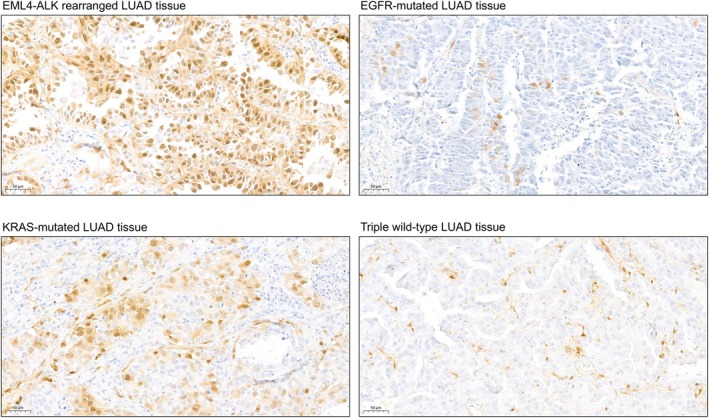
Immunohistochemistry staining of phospho‐p44/42 MAPK (Erk1/2) (Thr202/Tyr204) antibody to assess MAPK3/MAPK1 activity in LUAD tumors. Scale bar: 50 μm. Brown color represents positive staining.

**Fig. 10:**
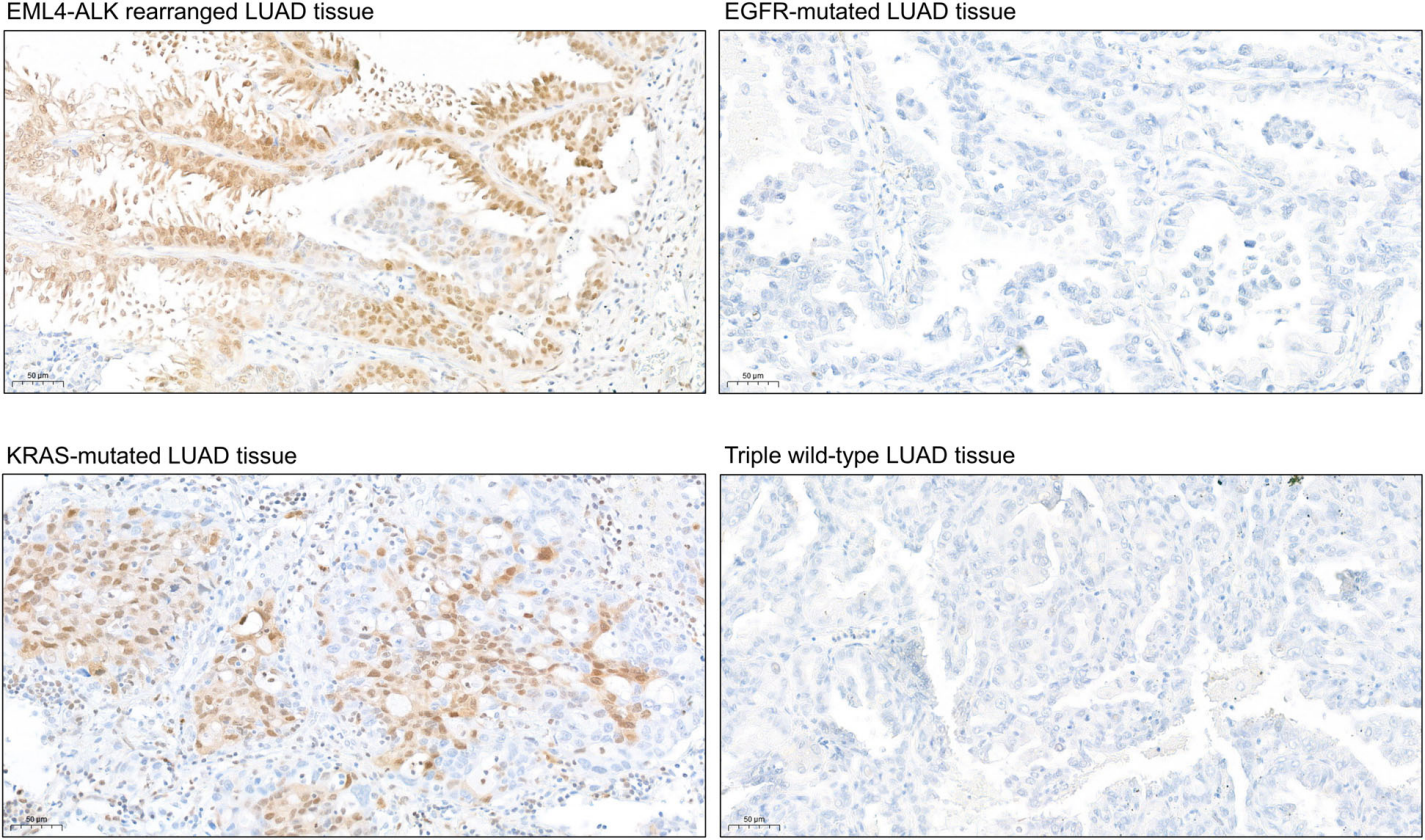
Immunohistochemistry staining of phospho‐p38 MAPK (Thr180/Tyr182) antibody to assess MAPK11 activity in LUAD tumors. Scale bar: 50 μm. Brown color represents positive staining.

Based on the results of KSEA and antibody availability, we performed immunohistochemistry (IHC) on serial tissue sections (2 μm thick) using the phospho‐p44/42 MAPK (Erk1/2) (Thr202/Tyr204) antibody to assess MAPK3/MAPK1 activity, the phospho‐CDK2 (Thr160) antibody for CDK2 activity, and the phospho‐p38 MAPK (Thr180/Tyr182) antibody for MAPK11 activity. Representative images of the stained tumor regions are presented in Figs 9 and 10 and Figs S19 and S20. Phospho‐p44/42 staining showed higher positivity in *EML4–ALK*‐rearranged and *KRAS*‐mutated samples. These results are consistent with our MS‐based phosphoproteomic findings, where MAPK1 activity was also higher in the *EML4–ALK* and *KRAS* samples compared to WT and in *KRAS* compared to *EGFR*. Additionally, MAPK3 activity was elevated in *KRAS* samples relative to both WT and *EGFR*, further supporting the observed staining patterns. Phospho‐p38 MAPK staining showed higher positivity in *KRAS*‐mutated samples, variable positivity among the samples within the *EML4–ALK* and WT sample groups, and negativity in the *EGFR*‐mutated samples. These results partly align with the MS analysis, where MAPK11 activity was suppressed in *EGFR*‐mutated samples compared to WT. However, elevated activity was observed in WT samples compared to *KRAS*‐mutated samples. In the case of phospho‐CDK2, we did not observe positive staining in most of the samples.

## 4 Discussion

In the present pilot study, lung adenocarcinomas harboring different genetic alterations were investigated by MS‐based proteomic and phosphoproteomic analysis. On‐surface digestion was performed on small, selected regions of FFPE tissue sections derived from patients with LUAD, followed by phosphopeptide enrichment. Differentially expressed proteins and their biological roles are discussed in detail and compared with existing literature. Phosphoproteins related to RNA splicing are also discussed. Finally, the results of the KSEA are interpreted.

### 4.1 Potential genetic alteration‐specific proteins in LUAD

Proteins significantly altered between samples with a specific genetic alteration can serve as potential markers for further development in diagnostic or therapeutic strategies. These proteins and their relationship with LUAD harboring genetic alterations are summarized in this section.

We identified lower levels of cathepsin L1 and elevated levels of 15‐hydroxyprostaglandin dehydrogenase (15‐PGDH) and stimulator of interferon genes protein (STING1) in *EML4–ALK*‐rearranged samples compared to the other three groups. Cathepsin L has a diverse role in cancer progression, promoting tumor invasion and metastatic activity. It has been identified as a downstream target of *KRAS*; moreover, the under‐expression of cathepsin L was detected in *KRAS*‐mutated, *EGFR*‐mutated, as well as in LUAD tumors without these mutations [50]. Additionally, the gene encoding cathepsin has been identified as a potential molecular marker for identifying *KRAS* mutations [51]. While the under‐expression of cathepsin L has been observed in *KRAS* and *EGFR*‐mutated tumors, we found it to be under‐expressed in *EML4–ALK*‐rearranged samples in our dataset, suggesting that its role may vary between different genetic alterations. This highlights the complexity of cathepsin L regulation in lung cancer, which requires further investigation to fully understand its contribution to tumor progression.

Although the elevated levels of 15‐PGDH and STING1 have not been previously associated with ALK alterations, both proteins have been implicated in other genetic mutations in lung cancer. 15‐PGDH was previously found to be under‐expressed in NSCLC tumors compared with matched normal samples; furthermore, inhibition of the *EGFR* pathway significantly elevated its expression levels [52]. Elevated expression of STING1 has been correlated with improved survival, early‐stage disease, and *EGFR* or *KRAS* mutations [53].

Moreover, our results showed that lysosome‐associated membrane glycoprotein 1 (LAMP1) was significantly under‐expressed in *EGFR* samples compared to the other genetic alterations or the WT samples. Previous studies have reported distinct subcellular localization patterns of LAMP1 depending on *EGFR* mutation status, with a more diffuse cytoplasmic distribution observed in *EGFR*‐mutant samples and a perinuclear localization in wild‐type cases [54]. Although this study did not directly compare expression levels, the altered localization was suggested as a potential diagnostic feature and a possible predictor of *EGFR* tyrosine kinase inhibitor response [55].

Additionally, nucleoside diphosphate kinase A was found to be under‐expressed in *EGFR* samples in the present study. However, although a direct link between the expressions of this protein and *EGFR* mutation has not been conclusively established, it has already been identified as a promising prognostic marker for NSCLC: Lower nucleoside diphosphate kinase A expression was strongly associated with increased risk of NSCLC, advanced TNM staging, poorer tumor differentiation, positive lymph node metastasis, lung adenocarcinoma, and a lower 5‐year overall survival rate in NSCLC patients [56].

### 4.2 Genetic alteration‐specific phosphorylation changes are enriched in RNA splicing‐related proteins

Functional enrichment analysis of 44 phosphoproteins with phosphosites potentially specific to a genetic alteration revealed an association with RNA splicing. Aberrant splicing is frequently observed in cancer, as changes in signaling affect the expression of regulatory splicing factors and the post‐translational modifications of these proteins [57]. Elevated spliceosome expression has been previously identified in human LUAD samples with *EGFR* mutations compared to non‐mutant *EGFR* samples [23]. In another study, where the effects of oncogenic *KRAS* on alternative splicing were investigated using human lung cell lines, alteration in phosphorylation of SRSF1, SRSF2, and SRSF7 proteins occurred in *KRAS* ^G12V^ and *KRAS* ^Q61H^ cells compared to *KRAS* wild‐type cells [58]. Our results showed that many SRSF phosphosites were altered. For example, a higher level of phosphorylation at SRSF1 S96 was observed in *EML4–ALK* and WT samples compared to *KRAS*‐mutated samples. SRSF1 S94 was identified as a potentially *KRAS*‐specific phosphosite, showing a lower level of phosphorylation. Additionally, SRRM2 is essential for organizing nuclear speckle formation, which is important for alternative splicing [59]. We identified several phosphosites of SRRM2 that showed potential alteration specificity, for example, S2694 for *EML4–ALK*, T1880, S1101 for *EGFR*, and S2702 for *KRAS*.

### 4.3 Kinase–substrate enrichment analysis

KSEA identified that the activities of 10 kinases were significantly altered between the four groups. Mitogen‐activated protein kinase 1 (MAPK1) showed suppressed activities in WT samples compared to samples with genetic alterations on *EML4–ALK* and *KRAS* and elevated activity in *KRAS*‐mutated samples compared to *EGFR*‐mutated samples. MAPK3 showed very similar activities compared to MAPK1, but it was not significantly altered in the comparison of *EML4–ALK* and WT. Additionally, the activities of MAPK7 and MAPK11 were suppressed in the WT samples compared to *EGFR*‐mutated and *KRAS*‐mutated samples. The MAPK pathway can be activated following the activation of RTKs, like *EGFR* [60]. Disturbance of the MAPK signaling in cancer is linked to uncontrolled cell proliferation and resistance to chemotherapy and targeted therapies [60]. Overexpression of the members of the MAPK pathway is known oncogenes in a large variety of tumors; they might be potential biomarkers for predicting the progression and prognosis of patients with NSCLC [61, 62]. Sousa et al. [22] found an increase in the predicted activities of kinases in the MAPK pathway for *KRAS* ^G12C^‐mutated samples.

In our results, cyclin‐dependent kinase 2 (CDK2) activity was elevated in *EML4–ALK* compared to the other sample groups. CDKs are serine/threonine kinases and major regulators of cell cycle progression, and dysregulation of CDK‐dependent pathways is often observed in NSCLC. Multiple CDK inhibitors, approved by the Food and Drug Administration (FDA), have garnered great interest in lung cancer, especially with the recent development of highly specific CDK4/6 inhibitors such as Palbociclib, Ribociclib, and Abemaciclib, which have shown promise in clinical trials for advanced metastatic breast cancer and are now being explored for lung cancer treatment [63]. Furthermore, CDK inhibition can bring improvements to the treatment of certain drug‐resistant *EML4–ALK* cells [64].

### 4.4 Limitations

Our exploratory study has considerable limitations. A relatively low number of samples was analyzed, and the starting sample amount was very low, imitating the size of small tissue biopsies. Tumor‐adjacent normal tissue areas were not investigated in the present cohort; only wild‐type, *EML4–ALK*‐rearranged, *KRAS*‐mutated, and *EGFR*‐mutated LUAD samples were compared. The validation of phosphosites potentially specific to a genetic alteration was not performed; however, immunohistochemical characterization of phospho‐p44/42 MAPK (Erk1/2) (Thr202/Tyr204), phospho‐CDK2 (Thr160), and phospho‐p38 MAPK (Thr180/Tyr182) was performed using parallel tissue sections. Additionally, phosphotyrosine sites were not enriched and hence were not detected in the analysis despite their importance in cell signaling. Moreover, since the cancerous cells were not sorted for analysis and the tissues are very heterogeneous, the identified differences can also be attributed to tumor heterogeneity.

## 5 Conclusions

In the present pilot study, we conducted an integrated proteomic and phosphoproteomic analysis on small regions of FFPE tissue sections to uncover biological processes and phosphorylation events specific to LUAD with and without *EML4–ALK* rearrangement, *EGFR* mutation, or *KRAS* mutation. Proteomic analysis revealed significant alterations in approximately one‐quarter of the investigated proteins, with *EGFR*‐mutated samples exhibiting a distinct protein expression pattern. Several proteins, such as cathepsin L and lysosome‐associated membrane glycoprotein 1, were identified as potentially specific to one of the genetic alterations. These proteins have previously been linked to *EGFR* or *KRAS* mutations and are known as prognostic markers for NSCLC and other cancers. Phosphoproteomic analysis uncovered significant differences in phosphosites among groups. Phosphoproteins with potential genetic alteration‐specific phosphosites were enriched in RNA splicing‐related processes. Kinase–substrate enrichment analysis indicated altered activities of 10 kinases, including MAPKs and CDKs. The results of this pilot study emphasize the need for further validation of the phosphosites and proteins potentially distinguishing the investigated LUAD types, as well as in‐depth investigations of these genetic alterations to enhance diagnostic and therapeutic strategies for LUAD.

## Conflict of interest

The authors declare no conflict of interest.

## Author contributions

FB and LT conceptualized and designed the study; ER, IL, TH, and JM collected the tissue samples and the patient data; FB and MB performed the sample preparation; GK and ZS performed the proteomic measurements; FB and SS analyzed the data; LV and IK performed the immunohistochemistry experiments; FB wrote the original draft; LT and LD provided funding, resources, and supervised the work. All authors were involved in correcting the initial draft and approving the manuscript draft.

## Supporting information


**Fig. S1.** GSEA dot plot of significantly enriched GOBP terms in EML4–ALK‐EGFR comparison, where the *x*‐axis represents the Normalized Enrichment Score (NES).
**Fig. S2.** GSEA dot plot of significantly enriched GOBP terms in EML4–ALK‐KRAS comparison, where the *x*‐axis represents the Normalized Enrichment Score (NES).
**Fig. S3.** GSEA dot plot of significantly enriched GOBP terms in EML4–ALK‐WT comparison, where the *x*‐axis represents the Normalized Enrichment Score (NES).
**Fig. S4.** GSEA dot plot of significantly enriched GOBP terms in EGFR‐KRAS comparison, where the *x*‐axis represents the Normalized Enrichment Score (NES).
**Fig. S5.** GSEA dot plot of significantly enriched GOBP terms in KRAS‐WT comparison, where the *x*‐axis represents the Normalized Enrichment Score (NES).
**Fig. S6.** GSEA plots of significantly enriched GOBP terms in EML4–ALK‐EGFR comparison.
**Fig. S7.** GSEA plots of significantly enriched GOBP terms in EGFR‐KRAS comparison.
**Fig. S8.** GSEA plots of significantly enriched GOBP terms in EGFR‐WT comparison.
**Fig. S9.** Correlation of significantly altered phosphorylation sites and corresponding protein expression changes in the EML4–ALK‐EGFR comparison.
**Fig. S10.** Correlation of significantly altered phosphorylation sites and corresponding protein expression changes in the EML4–ALK‐KRAS comparison.
**Fig. S11.** Correlation of significantly altered phosphorylation sites and corresponding protein expression changes in the EML4–ALK‐WT comparison.
**Fig. S12.** Correlation of significantly altered phosphorylation sites and corresponding protein expression changes in the EGFR‐KRAS comparison.
**Fig. S13.** Correlation of significantly altered phosphorylation sites and corresponding protein expression changes in the EGFR‐WT comparison.
**Fig. S14.** Correlation of significantly altered phosphorylation sites and corresponding protein expression changes in the KRAS‐WT comparison.
**Fig. S15.** Prediction of kinase‐substrate interactions based on the 183 altered phosphosites.
**Fig. S16.** Interaction network of the inferred kinases based on the 183 altered phosphosites.
**Fig. S17.** Visualization of functional enrichment analysis in STRING for GOCC terms of 44 phosphoproteins with potential alteration‐specific phosphosites.
**Fig. S18.** Visualization of functional enrichment analysis in STRING for GOMF terms of 44 phosphoproteins with potential alteration‐specific phosphosites.
**Fig. S19.** Immunohistochemistry staining of phospho‐CDK2 (Thr160) antibody to assess CDK2 activity in EML4–ALK‐rearranged LUAD tumor.
**Fig. S20.** Isotype control for immunohistochemistry analysis.

**Table S1.** Detailed information about the samples analyzed.

**Table S2.** Summary of the parameters used for all the software.

**Table S3.** List of 504 proteins along with the calculated *P*‐values and fold‐change values for the six different two‐group comparisons.

**Table S4.** The list of the enriched GOBP terms from the gene set enrichment analysis.

**Table S5.** List of 211 phosphosites along with the calculated *P*‐values and fold‐change values for the six different two‐group comparisons.

**Table S6.** The combined score of substrates and the predicted kinase‐substrate scores.

**Table S7.** The list of the enriched GO terms from the functional enrichment analysis of the 44 phosphoproteins with potential alteration‐specific phosphosites.

**Table S8.** Enriched kinases between the four sample groups based on KSEA of 202 kinase‐substrate pairs.

## Data Availability

Experimental data were submitted to the MassIVE data repository with the ID: MSV000095018. The data are available under the https://doi.org/10.25345/C5W37M669 link and can be downloaded via FTP (ftp://massive.ucsd.edu/v08/MSV000095018/). The manuscript contains Supporting Information.

## References

[1] Siegel RL , Miller KD , Fuchs HE , Jemal A . Cancer statistics, 2021. CA Cancer J Clin. 2021;71:7–33. 10.3322/caac.21654 33433946

[2] Siegel RL , Miller KD , Wagle NS , Jemal A . Cancer statistics, 2023. CA Cancer J Clin. 2023;73:17–48. 10.3322/caac.21763 36633525

[3] Inamura K . Lung cancer: understanding its molecular pathology and the 2015 WHO classification. Front Oncol. 2017;7:193. 10.3389/fonc.2017.00193 28894699 PMC5581350

[4] Kumar A , Kumar A . Non‐small‐cell lung cancer‐associated gene mutations and inhibitors. Adv Cancer Biol Metastasis. 2022;6:100076. 10.1016/j.adcanc.2022.100076

[5] Passaro A , Attili I , Rappa A , Vacirca D , Ranghiero A , Fumagalli C , et al. Genomic characterization of concurrent alterations in non‐small cell lung cancer (NSCLC) harboring actionable mutations. Cancer. 2021;13:2172. 10.3390/cancers13092172 PMC8124171 33946519

[6] Pikor LA , Ramnarine VR , Lam S , Lam WL . Genetic alterations defining NSCLC subtypes and their therapeutic implications. Lung Cancer. 2013;82:179–189. 10.1016/j.lungcan.2013.07.025 24011633

[7] Xiao Y , Liu P , Wei J , Zhang X , Guo J , Lin Y . Recent progress in targeted therapy for non‐small cell lung cancer. Front Pharmacol. 2023;14:1125547. 10.3389/fphar.2023.1125547 36909198 PMC9994183

[8] Colombino M , Paliogiannis P , Cossu A , Santeufemia DA , Pazzola A , Fadda GM , et al. EGFR, KRAS, BRAF, ALK, and cMET genetic alterations in 1440 Sardinian patients with lung adenocarcinoma. BMC Pulm Med. 2019;19:209. 10.1186/s12890-019-0964-x 31711449 PMC6849322

[9] Lee B , Lee T , Lee SH , Choi YL , Han J . Clinicopathologic characteristics of EGFR, KRAS, and ALK alterations in 6,595 lung cancers. Oncotarget. 2016;7:23874–23884. 10.18632/oncotarget.8074 26992209 PMC5029670

[10] Li P , Gao Q , Jiang X , Zhan Z , Yan Q , Li Z , et al. Comparison of Clinicopathological features and prognosis between ALK rearrangements and EGFR mutations in surgically resected early‐stage lung adenocarcinoma. J Cancer. 2019;10:61–71. 10.7150/jca.26947 30662526 PMC6329857

[11] Hondelink LM , Ernst SM , Atmodimedjo P , Cohen D , Wolf JL , Dingemans A‐MC , et al. Prevalence, clinical and molecular characteristics of early stage EGFR‐mutated lung cancer in a real‐life west‐European cohort: implications for adjuvant therapy. Eur J Cancer. 2023;181:53–61. 10.1016/j.ejca.2022.12.010 36638752

[12] McGuire AL , McConechy MK , Melosky BL , English JC , Choi JJ , Peng D , et al. The clinically actionable molecular profile of early versus late‐stage non‐small cell lung cancer, an individual age and sex propensity‐matched pair analysis. Curr Oncol. 2022;29:2630–2643. 10.3390/curroncol29040215 35448189 PMC9031556

[13] Russano M , Perrone G , Di Fazio GR , Galletti A , Citarella F , Santo V , et al. Uncommon EGFR mutations in non‐small‐cell lung cancer. Precision Cancer Med. 2022;5:30.

[14] Cekani E , Epistolio S , Dazio G , Cefalì M , Wannesson L , Frattini M , et al. Molecular biology and therapeutic perspectives for K‐Ras mutant non‐small cell lung cancers. Cancers (Basel). 2022;14:4103. 10.3390/cancers14174103 36077640 PMC9454753

[15] Barreca A , Lasorsa E , Riera L , Machiorlatti R , Piva R , Ponzoni M , et al. Anaplastic lymphoma kinase in human cancer. J Mol Endocrinol. 2011;47:R11–R23. 10.1530/JME-11-0004 21502284

[16] Abbasian MH , Ardekani AM , Sobhani N , Roudi R . The role of genomics and proteomics in lung cancer early detection and treatment. Cancers (Basel). 2022;14:5144. 10.3390/cancers14205144 36291929 PMC9600051

[17] Higgins L , Gerdes H , Cutillas PR . Principles of phosphoproteomics and applications in cancer research. Biochem J. 2023;480:403–420. 10.1042/BCJ20220220 36961757 PMC10212522

[18] Lehtiö J , Arslan T , Siavelis I , Pan Y , Socciarelli F , Berkovska O , et al. Proteogenomics of non‐small cell lung cancer reveals molecular subtypes associated with specific therapeutic targets and immune‐evasion mechanisms. Nat Cancer. 2021;2:1224–1242. 10.1038/s43018-021-00259-9 34870237 PMC7612062

[19] Soltis AR , Bateman NW , Liu J , Nguyen T , Franks TJ , Zhang X , et al. Proteogenomic analysis of lung adenocarcinoma reveals tumor heterogeneity, survival determinants, and therapeutically relevant pathways. Cell Rep Med. 2022;3:100819. 10.1016/j.xcrm.2022.100819 36384096 PMC9729884

[20] Gillette MA , Satpathy S , Cao S , Dhanasekaran SM , Vasaikar SV , Krug K , et al. Proteogenomic characterization reveals therapeutic vulnerabilities in lung adenocarcinoma. Cell. 2020;182:200–225.e235. 10.1016/j.cell.2020.06.013 32649874 PMC7373300

[21] Liu Z , Liu Y , Qian L , Jiang S , Gai X , Ye S , et al. A proteomic and phosphoproteomic landscape of KRAS mutant cancers identifies combination therapies. Mol Cell. 2021;81:4076–4090.e4078. 10.1016/j.molcel.2021.07.021 34375582

[22] Sousa A , Dugourd A , Memon D , Petursson B , Petsalaki E , Saez‐Rodriguez J , et al. Pan‐cancer landscape of protein activities identifies drivers of signalling dysregulation and patient survival. Mol Syst Biol. 2023;19(3):e10631. 10.15252/msb.202110631 36688815 PMC9996241

[23] Xu J‐Y , Zhang C , Wang X , Zhai L , Ma Y , Mao Y , et al. Integrative proteomic characterization of human lung adenocarcinoma. Cell. 2020;182:245–261.e217. 10.1016/j.cell.2020.05.043 32649877

[24] Sugár SN , Molnár BA , Bugyi F , Kecskeméti G , Szabó Z , Laczó I , et al. Glycoproteomics Analysis of triple wild‐type lung adenocarcinoma tissue samples. J Proteome Res. 2025;24:2419–2429. 10.1021/acs.jproteome.4c01063 40175289 PMC12053933

[25] Reiniger L , Téglási V , Pipek O , Rojkó L , Glasz T , Vágvölgyi A , et al. Tumor necrosis correlates with PD‐L1 and PD‐1 expression in lung adenocarcinoma. Acta Oncol. 2019;58:1087–1094. 10.1080/0284186x.2019.1598575 31002007

[26] Smuk G , Pajor G , Szuhai K , Morreau H , Kocsmár I , Kocsmár É , et al. Attenuated isolated 3′ signal: a highly challenging therapy relevant ALK FISH pattern in NSCLC. Lung Cancer. 2020;143:80–85. 10.1016/j.lungcan.2020.03.007 32272316

[27] Sugár S , Tóth G , Bugyi F , Vékey K , Karászi K , Drahos L , et al. Alterations in protein expression and site‐specific N‐glycosylation of prostate cancer tissues. Sci Rep. 2021;11:15886. 10.1038/s41598-021-95417-5 34354152 PMC8342536

[28] Tóth G , Bugyi F , Sugár S , Mitulović G , Vékey K , Turiák L , et al. Selective TiO_2_ Phosphopeptide enrichment of complex samples in the Nanogram range. Separations. 2020;7:74.

[29] Bugyi F , Tóth G , Kovács KB , Drahos L , Turiák L . Comparison of solid‐phase extraction methods for efficient purification of phosphopeptides with low sample amounts. J Chromatogr A. 2022;1685:463597. 10.1016/j.chroma.2022.463597 36371923

[30] Bern M , Kil YJ , Becker C . Byonic: advanced peptide and protein identification software. Curr Protoc Bioinformatics. 2012;40:13.20.11–13.20.14. 10.1002/0471250953.bi1320s40 PMC3545648 23255153

[31] Tyanova S , Temu T , Cox J . The MaxQuant computational platform for mass spectrometry‐based shotgun proteomics. Nat Protoc. 2016;11:2301–2319. 10.1038/nprot.2016.136 27809316

[32] Ihaka R , Gentleman R . R: a language for data analysis and graphics. J Comput Graph Stat. 1996;5:299–314. 10.1080/10618600.1996.10474713

[33] RStudio Team . RStudio: integrated development for R. Boston, MA: RStudio, PBC; 2020. http://www.rstudio.com/

[34] Szklarczyk D , Kirsch R , Koutrouli M , Nastou K , Mehryary F , Hachilif R , et al. The STRING database in 2023: protein‐protein association networks and functional enrichment analyses for any sequenced genome of interest. Nucleic Acids Res. 2023;51:D638–D646. 10.1093/nar/gkac1000 36370105 PMC9825434

[35] Casado P , Rodriguez‐Prados J‐C , Cosulich SC , Guichard S , Vanhaesebroeck B , Joel S , et al. Kinase‐substrate enrichment Analysis provides insights into the heterogeneity of signaling pathway activation in leukemia cells. Sci Signal. 2013;6:rs6. 10.1126/scisignal.2003573 23532336

[36] Wiredja DD , Koyutürk M , Chance MR . The KSEA app: a web‐based tool for kinase activity inference from quantitative phosphoproteomics. Bioinformatics. 2017;33:3489–3491. 10.1093/bioinformatics/btx415 28655153 PMC5860163

[37] Kowarik A , Templ M . Imputation with the R package VIM. J Stat Softw. 2016;74:1–16. 10.18637/jss.v074.i07

[38] Kim HJ , Kim T , Hoffman NJ , Xiao D , James DE , Humphrey SJ , et al. PhosR enables processing and functional analysis of phosphoproteomic data. Cell Rep. 2021;34:108771. 10.1016/j.celrep.2021.108771 33626354

[39] Wickham H . ggplot2: elegant graphics for data analysis. New York, NY: Springer‐Verlag New York; 2016.

[40] John CR , Watson D , Russ D , Goldmann K , Ehrenstein M , Pitzalis C , et al. M3C: Monte Carlo reference‐based consensus clustering. Sci Rep. 2020;10(1):1816. 10.1038/s41598-020-58766-1 32020004 PMC7000518

[41] Zhao S , Guo Y , Sheng Q , Shyr Y . Heatmap3: an improved heatmap package with more powerful and convenient features. BMC Bioinformatics. 2014;15(Suppl 10):P16. 10.1186/1471-2105-15-S10-P16

[42] Gatto L , Lilley KS . MSnbase‐an R/Bioconductor package for isobaric tagged mass spectrometry data visualization, processing and quantitation. Bioinformatics. 2012;28:288–289. 10.1093/bioinformatics/btr645 22113085

[43] Xu S , Hu E , Cai Y , Xie Z , Luo X , Zhan L , et al. Using clusterProfiler to characterize multiomics data. Nat Protoc. 2024;19:3292–3320. 10.1038/s41596-024-01020-z 39019974

[44] Hornbeck PV , Zhang B , Murray B , Kornhauser JM , Latham V , Skrzypek E . PhosphoSitePlus, 2014: mutations, PTMs and recalibrations. Nucleic Acids Res. 2015;43:D512–D520. 25514926 10.1093/nar/gku1267 PMC4383998

[45] Linding R , Jensen LJ , Pasculescu A , Olhovsky M , Colwill K , Bork P , et al. NetworKIN: a resource for exploring cellular phosphorylation networks. Nucleic Acids Res. 2008;36:D695–D699. 10.1093/nar/gkm902 17981841 PMC2238868

[46] Cheng X . A comprehensive review of HER2 in cancer biology and therapeutics. Genes. 2024;15:903. 39062682 10.3390/genes15070903 PMC11275319

[47] Lin C‐C , Suen KM , Stainthorp A , Wieteska L , Biggs GS , Leitão A , et al. Targeting the Shc‐EGFR interaction with indomethacin inhibits MAP kinase pathway signalling. Cancer Lett. 2019;457:86–97. 10.1016/j.canlet.2019.05.008 31100409 PMC6584941

[48] Gregory MD , Ofosu‐Asante K , Lazarte JMS , Puente PE , Tawfeeq N , Belony N , et al. Treatment of a mutant KRAS lung cancer cell line with polyisoprenylated cysteinyl amide inhibitors activates the MAPK pathway, inhibits cell migration and induces apoptosis. PLoS One. 2024;19:e0312563. 10.1371/journal.pone.0312563 39436906 PMC11495567

[49] Wei C . The multifaceted roles of matrix metalloproteinases in lung cancer. Front Oncol. 2023;13:1195426. 10.3389/fonc.2023.1195426 37766868 PMC10520958

[50] Okudela K , Mitsui H , Woo T , Arai H , Suzuki T , Matsumura M , et al. Alterations in cathepsin L expression in lung cancers. Pathol Int. 2016;66:386–392. 10.1111/pin.12424 27327955

[51] Zhang J , Hu H , Xu S , Jiang H , Zhu J , Qin E , et al. The functional effects of key driver KRAS mutations on gene expression in lung cancer. Front Genet. 2020;11:17. 10.3389/fgene.2020.00017 32117436 PMC7010953

[52] Yang L , Amann JM , Kikuchi T , Porta R , Guix M , Gonzalez A , et al. Inhibition of epidermal growth factor receptor signaling elevates 15‐Hydroxyprostaglandin dehydrogenase in non–small‐cell lung cancer. Cancer Res. 2007;67:5587–5593. 10.1158/0008-5472.CAN-06-2287 17575121

[53] Lohinai Z , Dora D , Caldwell C , Rivard CJ , Suda K , Yu H , et al. Loss of STING expression is prognostic in non–small cell lung cancer. J Surg Oncol. 2022;125:1042–1052. 10.1002/jso.26804 35099823 PMC9304565

[54] Chin T‐M , Boopathy GTK , Man EPS , Clohessy JG , Csizmadia E , Quinlan MP , et al. Targeting microtubules sensitizes drug resistant lung cancer cells to lysosomal pathway inhibitors. Theranostics. 2020;10:2727–2743. 32194831 10.7150/thno.38729 PMC7052910

[55] Agarwal AK , Srinivasan N , Godbole R , More SK , Budnar S , Gude RP , et al. Role of tumor cell surface lysosome‐associated membrane protein‐1 (LAMP1) and its associated carbohydrates in lung metastasis. J Cancer Res Clin Oncol. 2015;141:1563–1574. 10.1007/s00432-015-1917-2 25614122 PMC11823972

[56] Min SH , Zheng QQ . Clinicopathological and prognostic significance of NM23 expression in patients with non‐small cell lung cancer: a systematic review and meta‐analysis. Medicine (Baltimore). 2021;100:e27919. 10.1097/md.0000000000027919 34964763 PMC8615335

[57] Gonçalves V , Pereira JFS , Jordan P . Signaling pathways driving aberrant splicing in cancer cells. Genes. 2017;9:9. 10.3390/genes9010009 29286307 PMC5793162

[58] Lo A , McSharry M , Berger AH . Oncogenic KRAS alters splicing factor phosphorylation and alternative splicing in lung cancer. BMC Cancer. 2022;22:1315. 10.1186/s12885-022-10311-1 36522653 PMC9756471

[59] Xu S , Lai S‐K , Sim DY , Ang Warren Shou L , Li HY , Roca X . SRRM2 organizes splicing condensates to regulate alternative splicing. Nucleic Acids Res. 2022;50:8599–8614. 10.1093/nar/gkac669 35929045 PMC9410892

[60] Cordover E , Minden A . Signaling pathways downstream to receptor tyrosine kinases: targets for cancer treatment. J Cancer Metastasis Treat. 2020;6:45. 10.20517/2394-4722.2020.101

[61] Alsharoh H , Chiroi P , Isachesku E , Tanasa RA , Pop O‐L , Pirlog R , et al. Personalizing therapy outcomes through mitogen‐activated protein kinase pathway inhibition in non‐small cell lung cancer. Biomedicine. 2024;12:1489. 10.3390/biomedicines12071489 PMC11275062 39062063

[62] Pradhan R , Singhvi G , Dubey SK , Gupta G , Dua K . MAPK pathway: a potential target for the treatment of non‐small‐cell lung carcinoma. Future Med Chem. 2019;11:793–795. 10.4155/fmc-2018-0468 30994024

[63] Zhang J , Xu D , Zhou Y , Zhu Z , Yang X . Mechanisms and implications of CDK4/6 inhibitors for the treatment of NSCLC. Front Oncol. 2021;11:676041. 10.3389/fonc.2021.676041 34395246 PMC8361448

[64] Paliouras AR , Buzzetti M , Shi L , Donaldson IJ , Magee P , Sahoo S , et al. Vulnerability of drug‐resistant EML4‐ALK rearranged lung cancer to transcriptional inhibition. EMBO Mol Med. 2020;12:e11099. 10.15252/emmm.201911099 32558295 PMC7338803

[65] Héder M , Rigó E , Medgyesi D , Lovas R , Tenczer S , Török F , et al. The past, present and future of the ELKH clouds. InfTars. 2022;22:128–137.

