# Supplementary material for: Unveiling unique protein and phosphorylation signatures in lung adenocarcinomas with and without ALK, EGFR, and KRAS genetic alterations
Source: Mol Oncol. 2025 Jul 7;19(11):3243–65. doi: 10.1002/1878-0261.70091 (PMC12591317; doi:10.1002/1878-0261.70091)
Supplement: Supplementary file 1 — Fig. S1. GSEA dot plot of significantly enriched GOBP terms in EML4–ALK‐EGFR comparison, where the x‐axis represents the Normalized Enrichment Score (NES). Fig. S2. GSEA dot plot of significantly enriched GOBP terms in EML4–ALK‐KRAS comparison, where the x‐axis represents the Normalized Enrichment Score (NES). Fig. S3. GSEA dot plot of significantly enriched GOBP terms in EML4–ALK‐WT comparison, where the x‐axis represents the Normalized Enrichment Score (NES). Fig. S4. GSEA dot plot of significantly enriched GOBP terms in EGFR‐KRAS comparison, where the x‐axis represents the Normalized Enrichment Score (NES). Fig. S5. GSEA dot plot of significantly enriched GOBP terms in KRAS‐WT comparison, where the x‐axis represents the Normalized Enrichment Score (NES). Fig. S6. GSEA plots of significantly enriched GOBP terms in EML4–ALK‐EGFR comparison. Fig. S7. GSEA plots of significantly enriched GOBP terms in EGFR‐KRAS comparison. Fig. S8. GSEA plots of significantly enriched GOBP terms in EGFR‐WT comparison. Fig. S9. Correlation of significantly altered phosphorylation sites and corresponding protein expression changes in the EML4–ALK‐EGFR comparison. Fig. S10. Correlation of significantly altered phosphorylation sites and corresponding protein expression changes in the EML4–ALK‐KRAS comparison. Fig. S11. Correlation of significantly altered phosphorylation sites and corresponding protein expression changes in the EML4–ALK‐WT comparison. Fig. S12. Correlation of significantly altered phosphorylation sites and corresponding protein expression changes in the EGFR‐KRAS comparison. Fig. S13. Correlation of significantly altered phosphorylation sites and corresponding protein expression changes in the EGFR‐WT comparison. Fig. S14. Correlation of significantly altered phosphorylation sites and corresponding protein expression changes in the KRAS‐WT comparison. Fig. S15. Prediction of kinase‐substrate interactions based on the 183 altered phosphosites. Fig. S16. Interaction networ [file MOL2-19-3243-s005.pdf]

Supplementary Figures

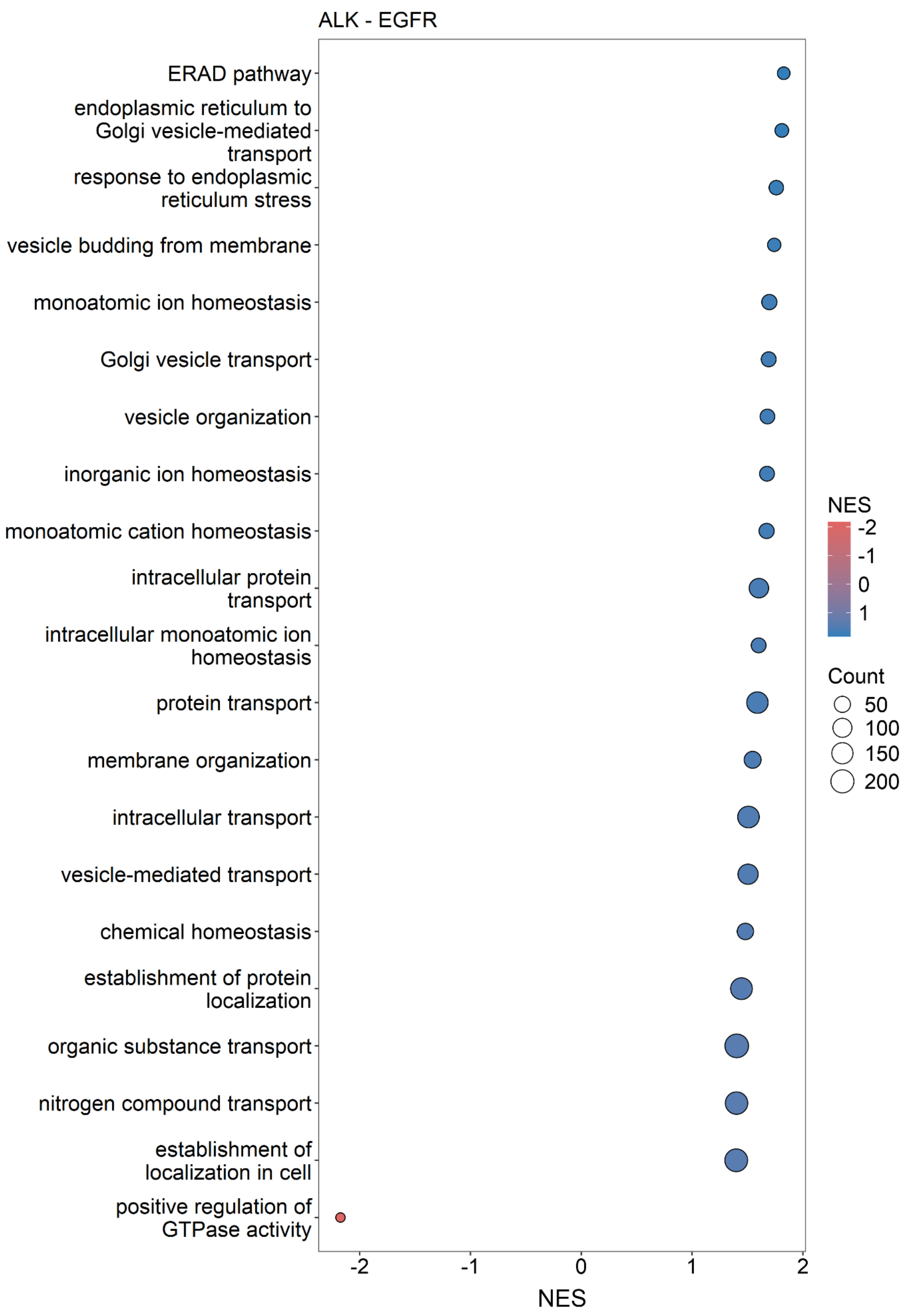

Figure S1. GSEA dot plot of significantly enriched GOBP terms in EML4–ALK-EGFR comparison, where the x-axis represents the Normalized Enrichment Score (NES). The size of the dots corresponds to the number of genes associated with the term, and the color represents the NES.

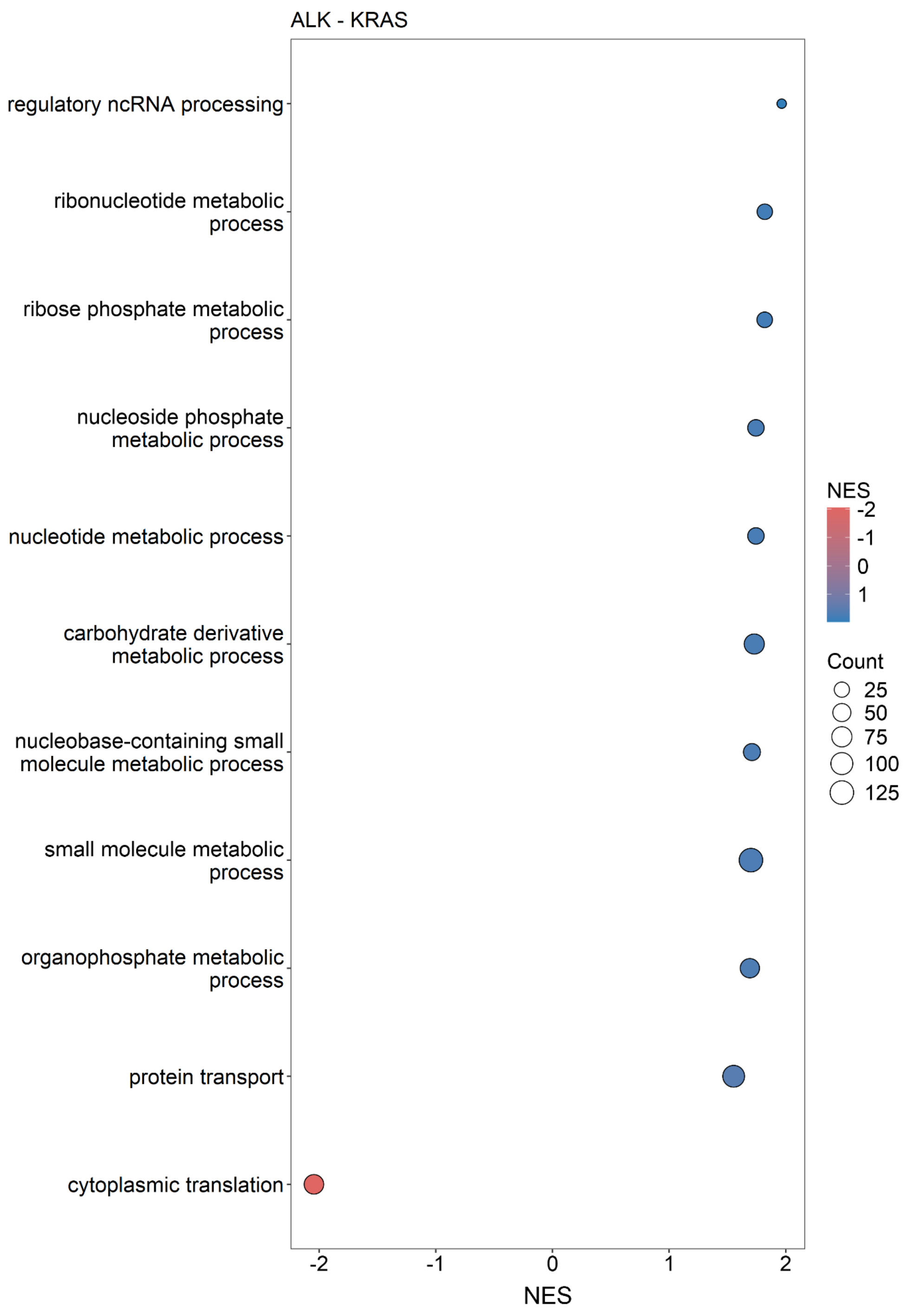

Figure S2. GSEA dot plot of significantly enriched GOBP terms in EML4–ALK-KRAS comparison, where the x-axis represents the Normalized Enrichment Score (NES). The size of the dots corresponds to the number of genes associated with the term, and the color represents the NES.

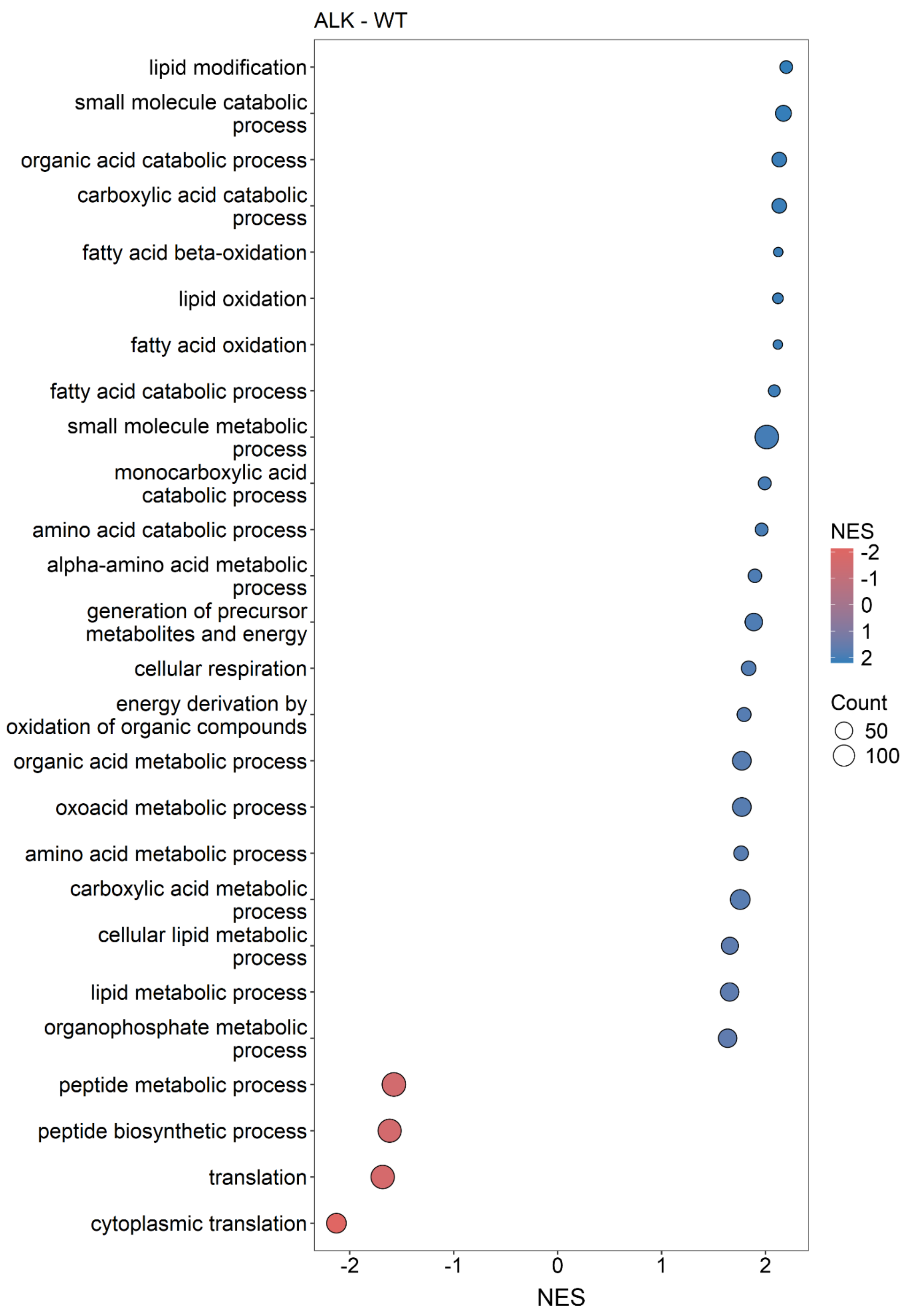

Figure S3. GSEA dot plot of significantly enriched GOBP terms in EML4–ALK-WT comparison, where the x-axis represents the Normalized Enrichment Score (NES). The size of the dots corresponds to the number of genes associated with the term, and the color represents the NES.

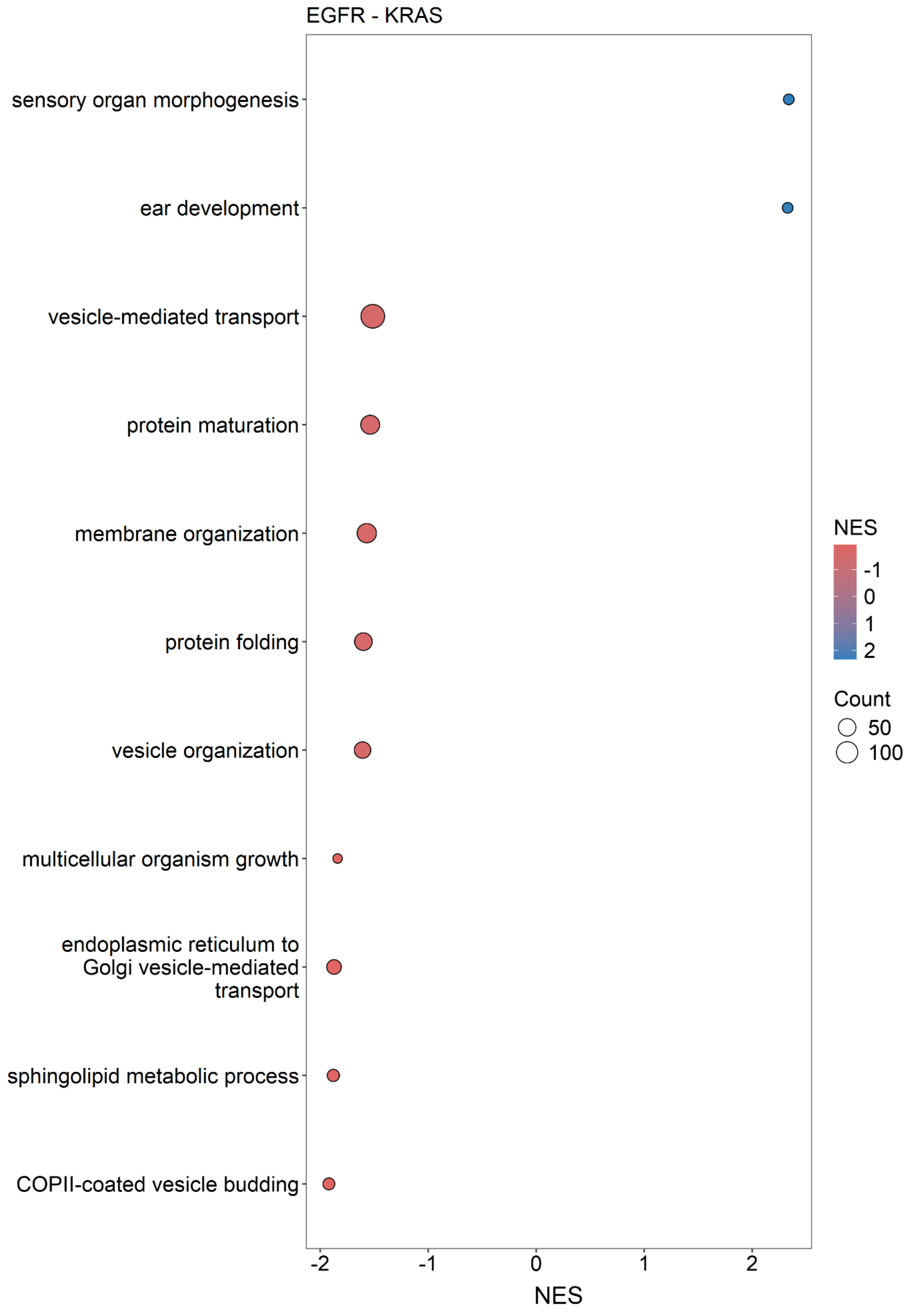

Figure S4. GSEA dot plot of significantly enriched GOBP terms in EGFR-KRAS comparison, where the x-axis represents the Normalized Enrichment Score (NES). The size of the dots corresponds to the number of genes associated with the term, and the color represents the NES.

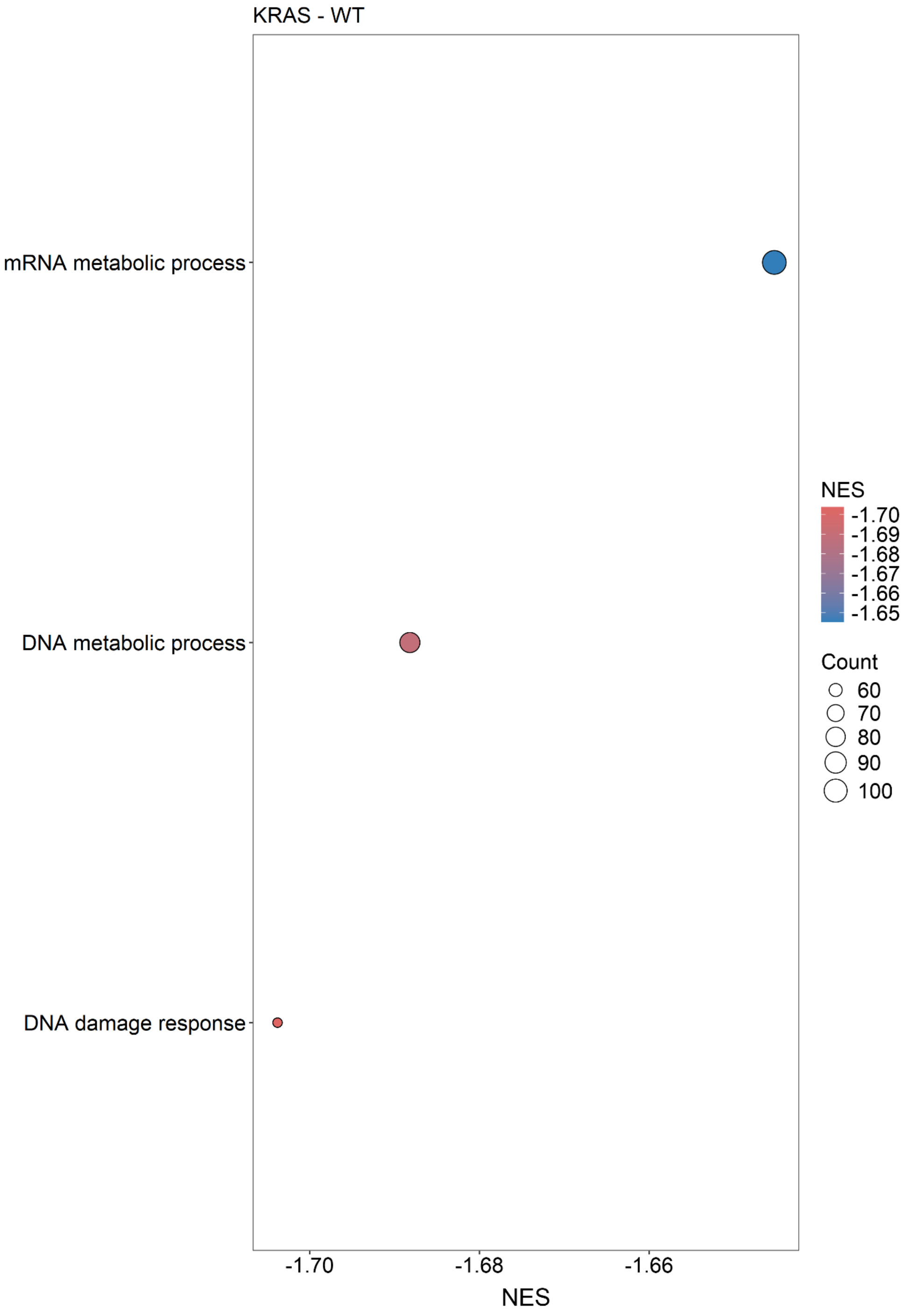

Figure S5. GSEA dot plot of significantly enriched GOBP terms in KRAS-WT comparison, where the x-axis represents the Normalized Enrichment Score (NES). The size of the dots corresponds to the number of genes associated with the term, and the color represents the NES.

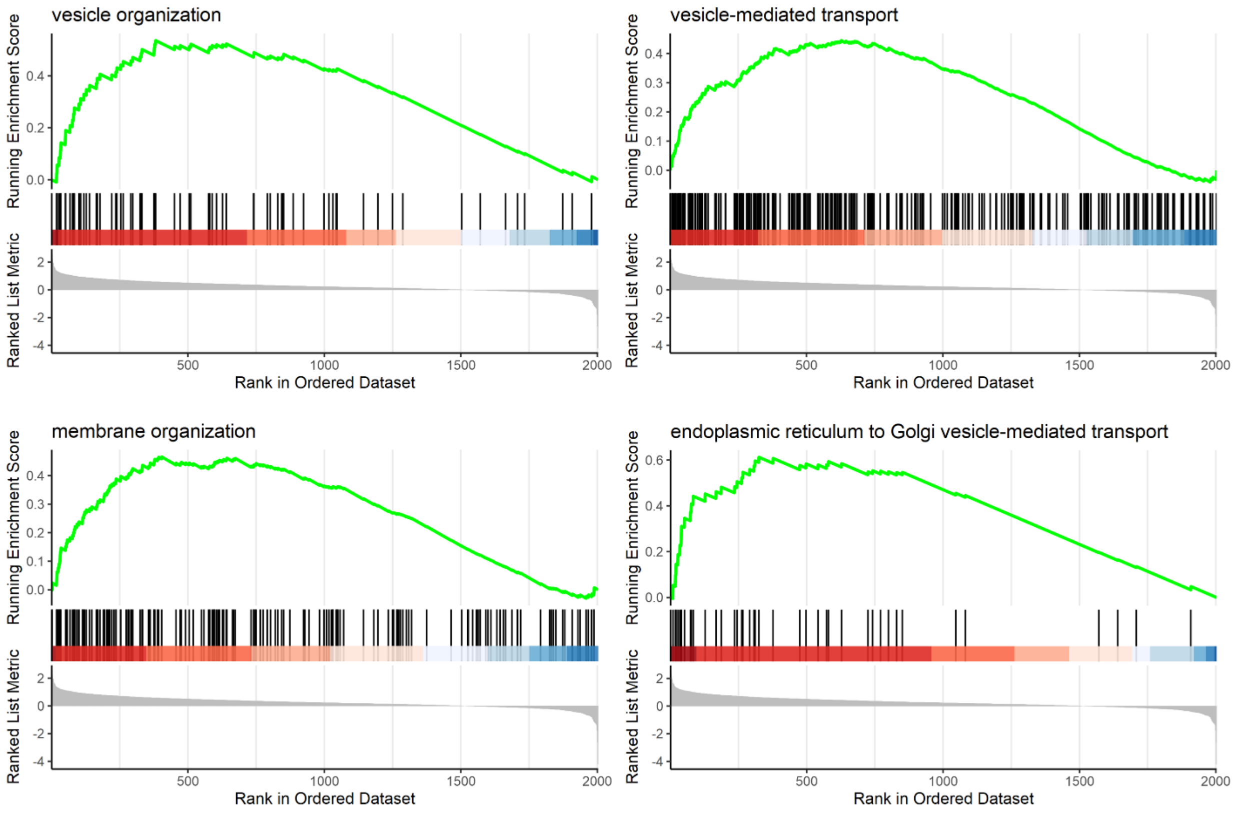

Figure S6. GSEA plots of significantly enriched GOBP terms in EML4–ALK-EGFR comparison. The x-axis represents the ranked dataset, while the y-axis shows the running enrichment score (green line), indicating the accumulation of genes associated with each term. The color gradient represents the ranked list metric, with red indicating genes more highly expressed in EML4–ALK-rearranged samples and blue indicating those more expressed in EGFR-mutated samples.

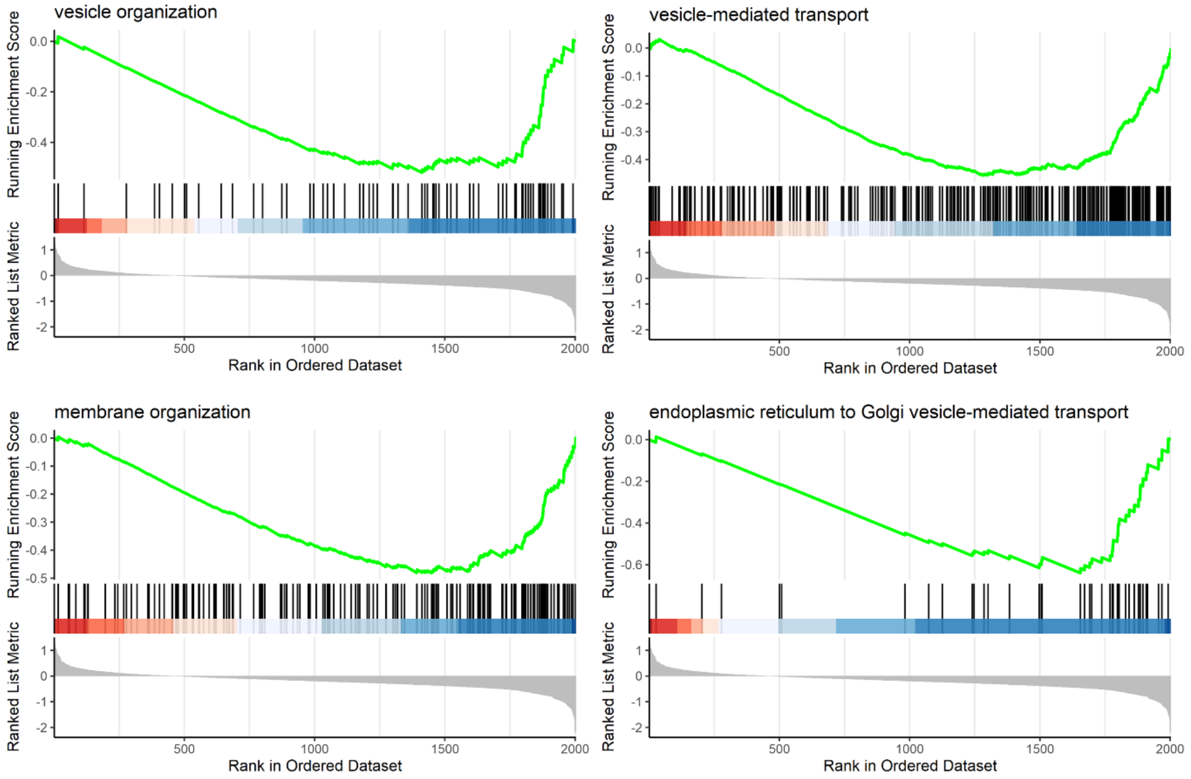

Figure S7. GSEA plots of significantly enriched GOBP terms in EGFR-KRAS comparison. The x-axis represents the ranked dataset, while the y-axis shows the running enrichment score (green line), indicating the accumulation of genes associated with each term. The color gradient represents the ranked list metric, with red indicating genes more highly expressed in EGFR-mutated samples and blue indicating those more expressed in KRAS-mutated samples.

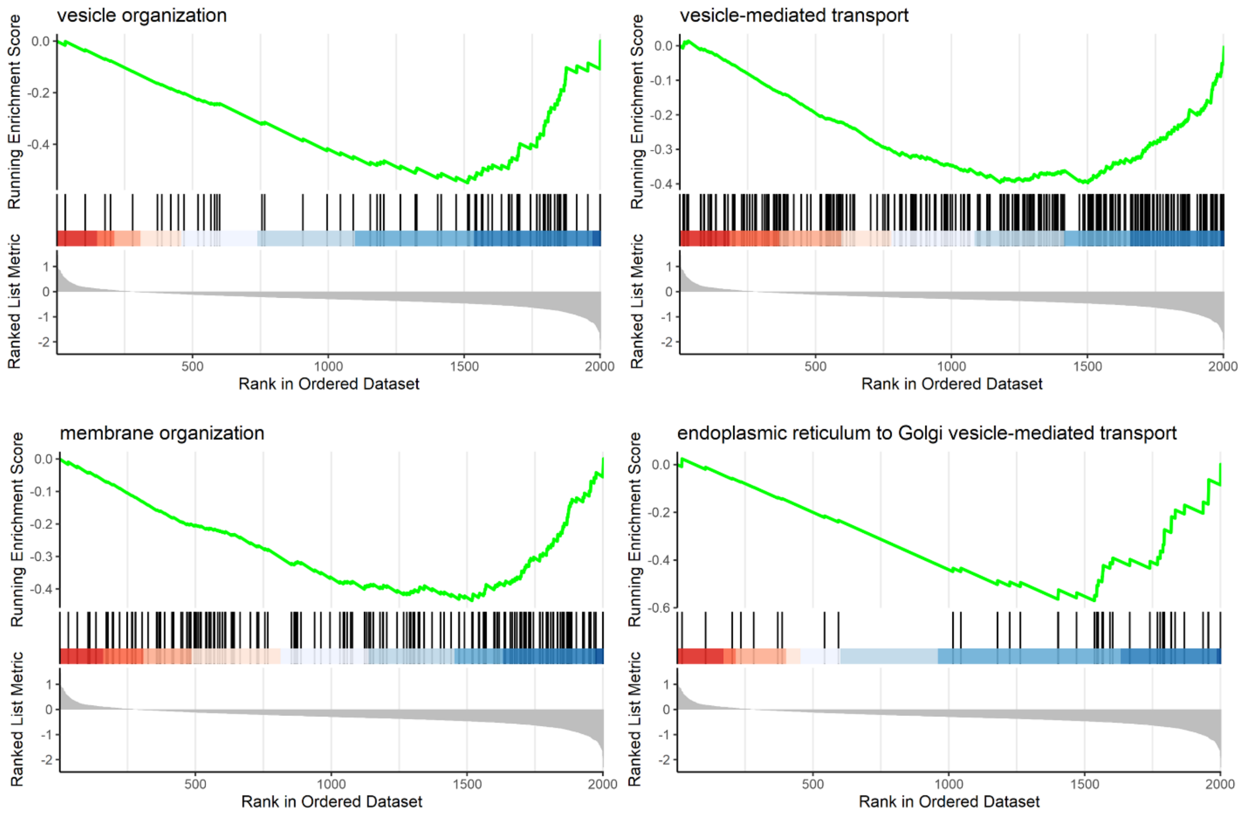

Figure S8. GSEA plots of significantly enriched GOBP terms in EGFR-WT comparison. The x-axis represents the ranked dataset, while the y-axis shows the running enrichment score (green line), indicating the accumulation of genes associated with each term. The color gradient represents the ranked list metric, with red indicating genes more highly expressed in EGFR-mutated samples and blue indicating those more expressed in WT samples.

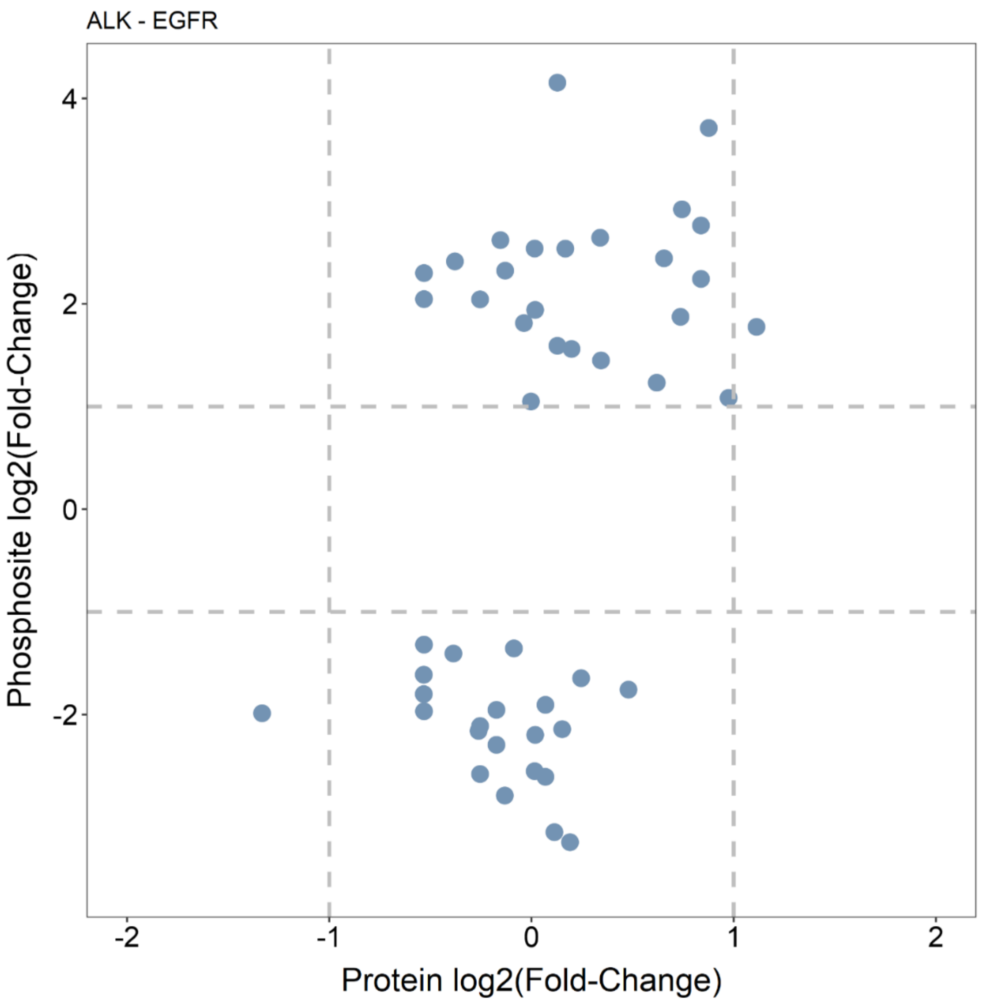

Figure S9. Correlation of significantly altered phosphorylation sites and corresponding protein expression changes in the EML4–ALK-EGFR comparison.

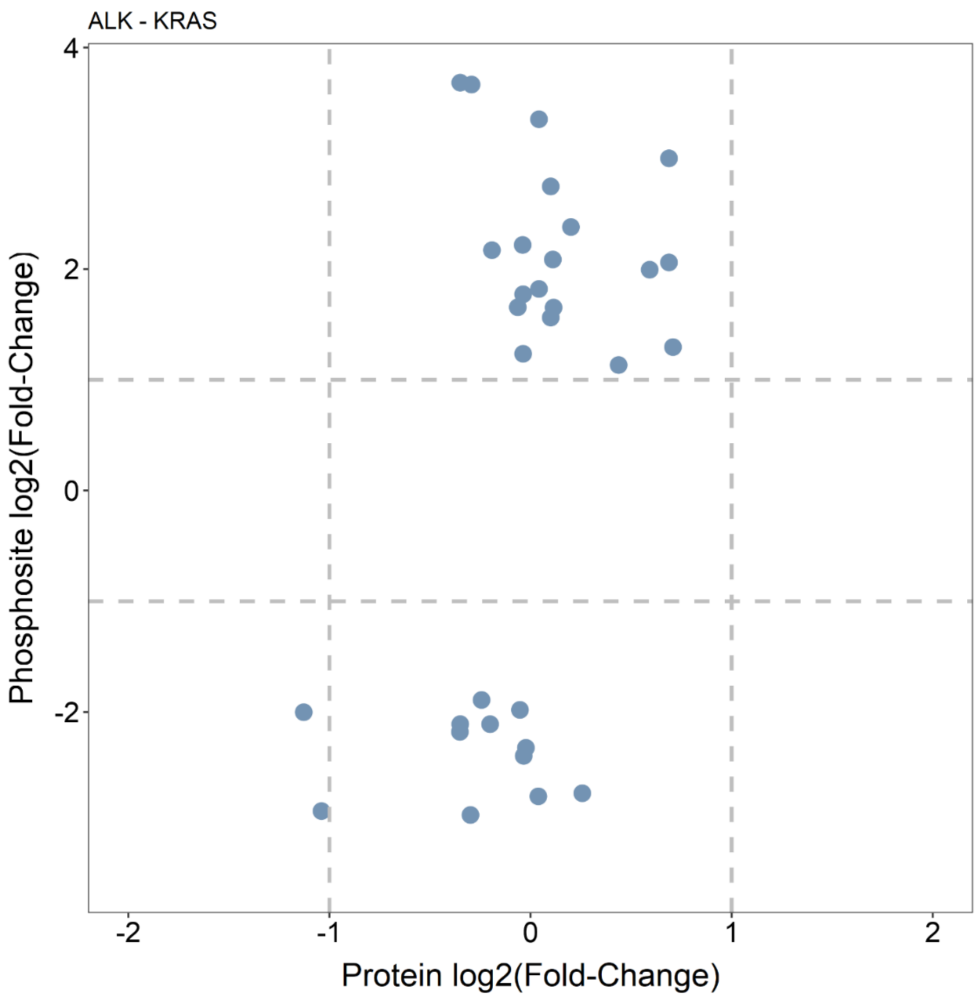

Figure S10. Correlation of significantly altered phosphorylation sites and corresponding protein expression changes in the EML4–ALK-KRAS comparison.

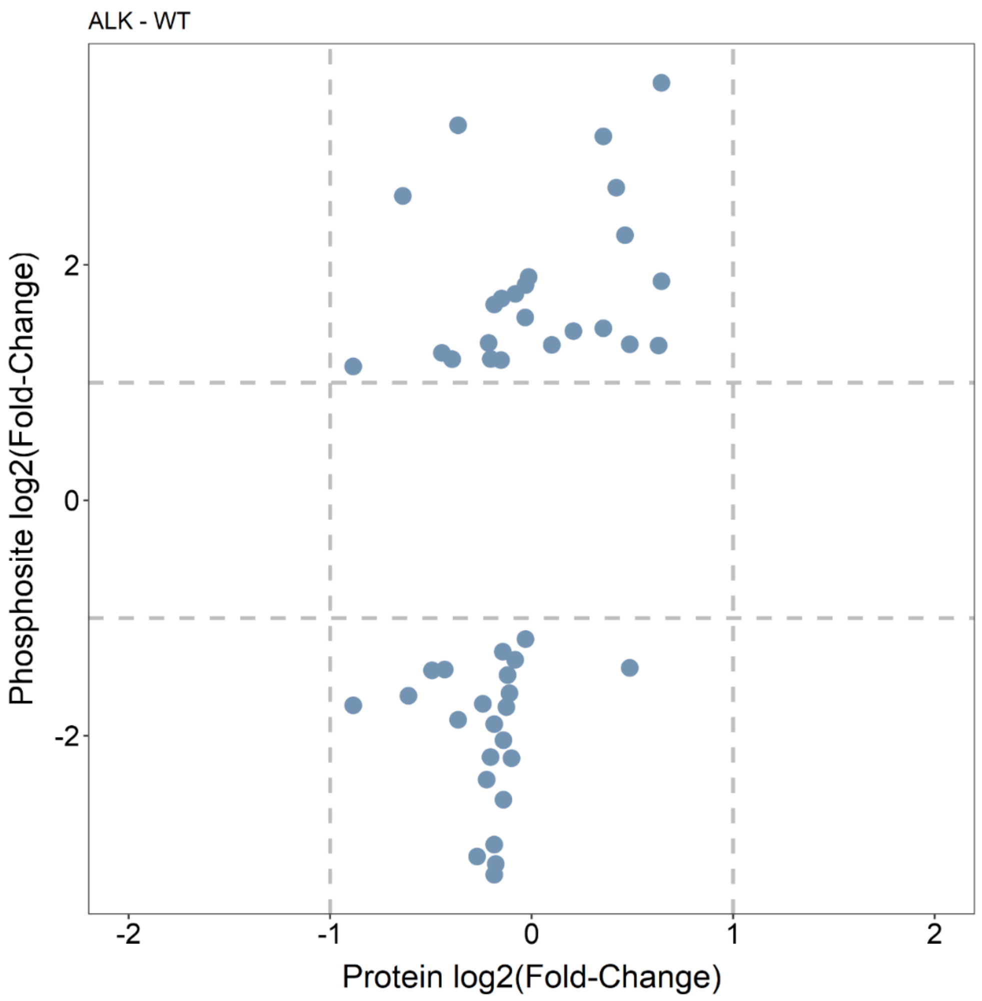

Figure S11. Correlation of significantly altered phosphorylation sites and corresponding protein expression changes in the EML4–ALK-WT comparison.

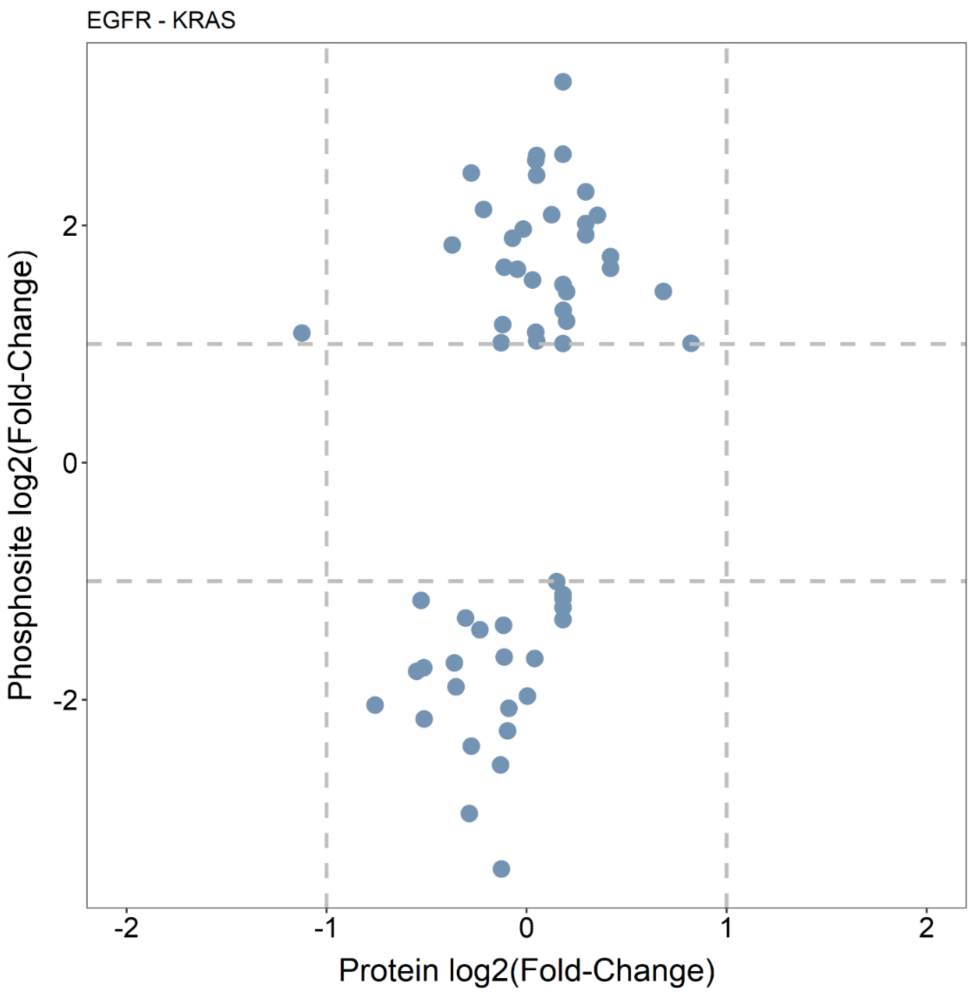

Figure S12. Correlation of significantly altered phosphorylation sites and corresponding protein expression changes in the EGFR-KRAS comparison.

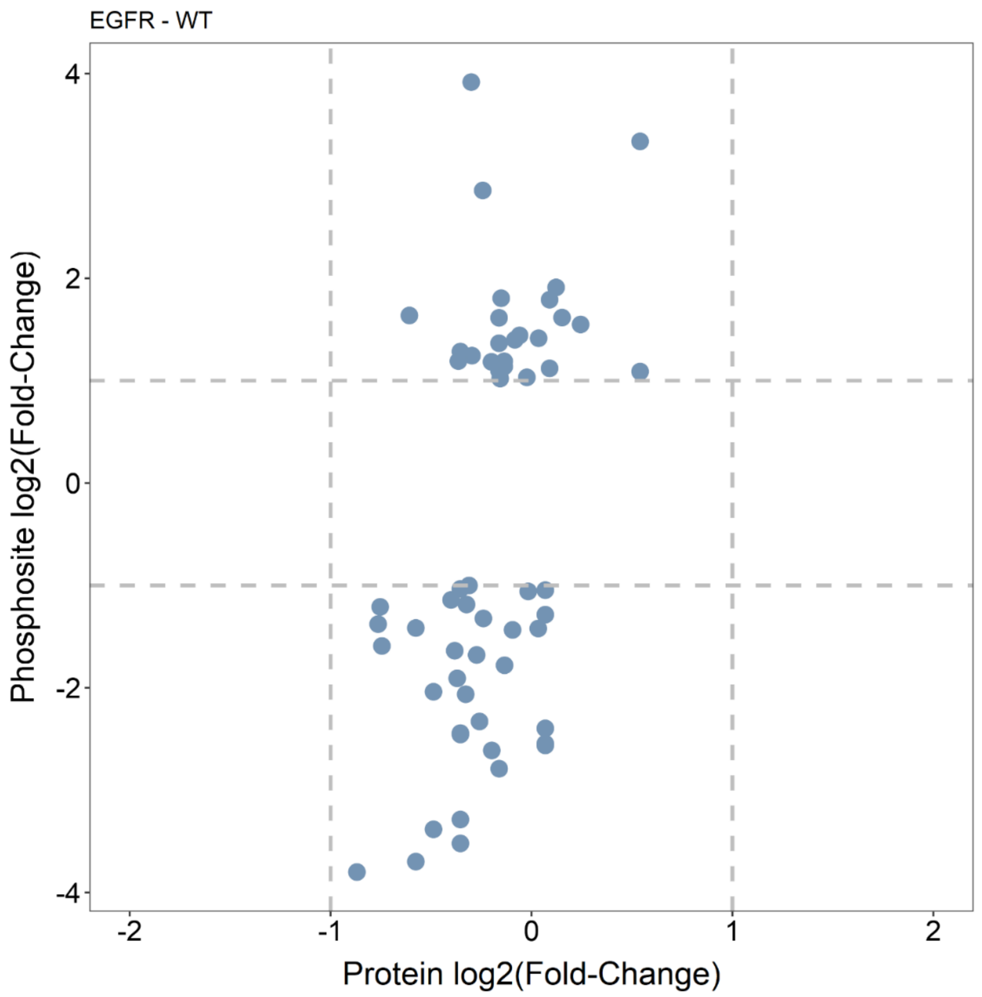

Figure S13. Correlation of significantly altered phosphorylation sites and corresponding protein expression changes in the EGFR-WT comparison.

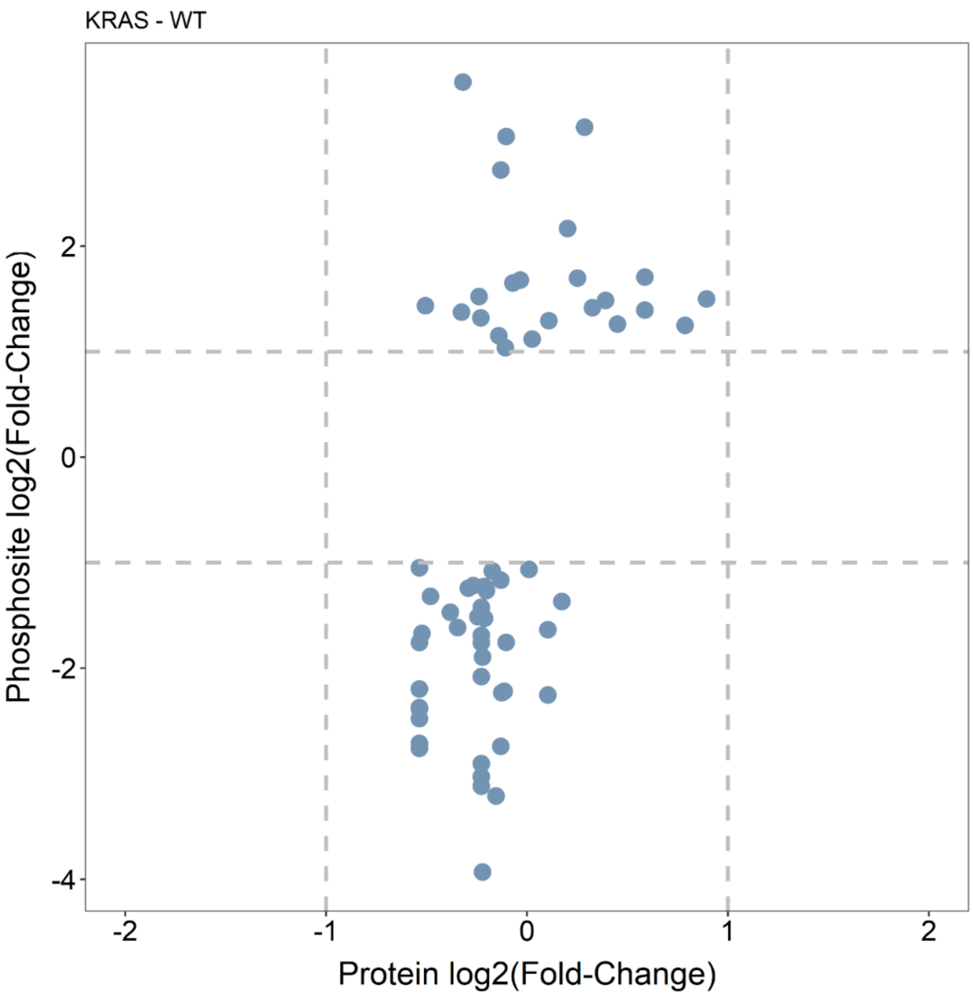

Figure S14. Correlation of significantly altered phosphorylation sites and corresponding protein expression changes in the KRAS-WT comparison.

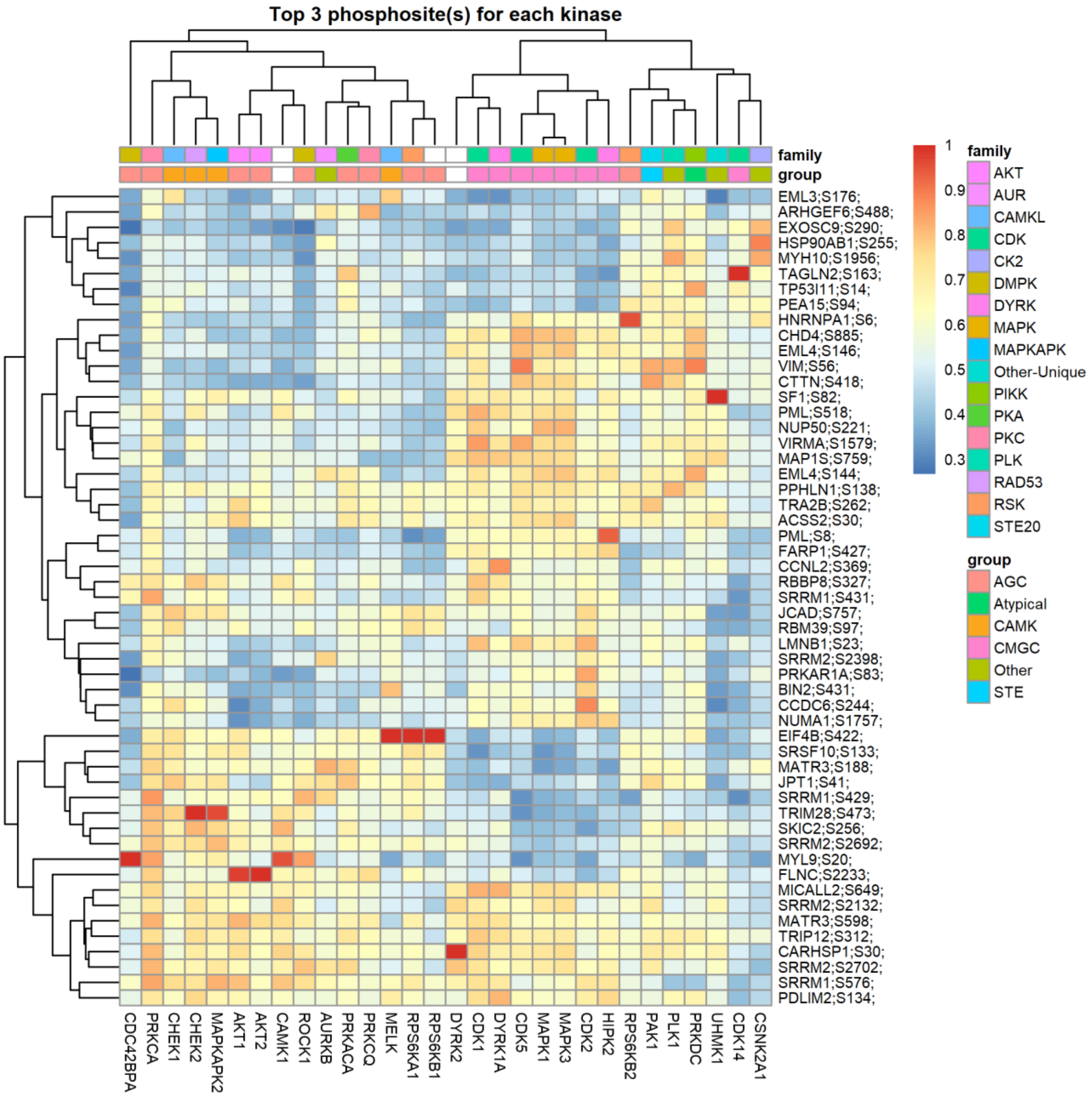

Figure S15. Prediction of kinase-substrate interactions based on the 183 altered phosphosites. Clustered heatmap showing predicted kinase-substrate interactions, kinases are color-coded based on their family and group. The combined score was calculated using PhosR, a higher score donates a better fit to a kinase motif and kinase-substrate phosphorylation profile of a phosphosite.

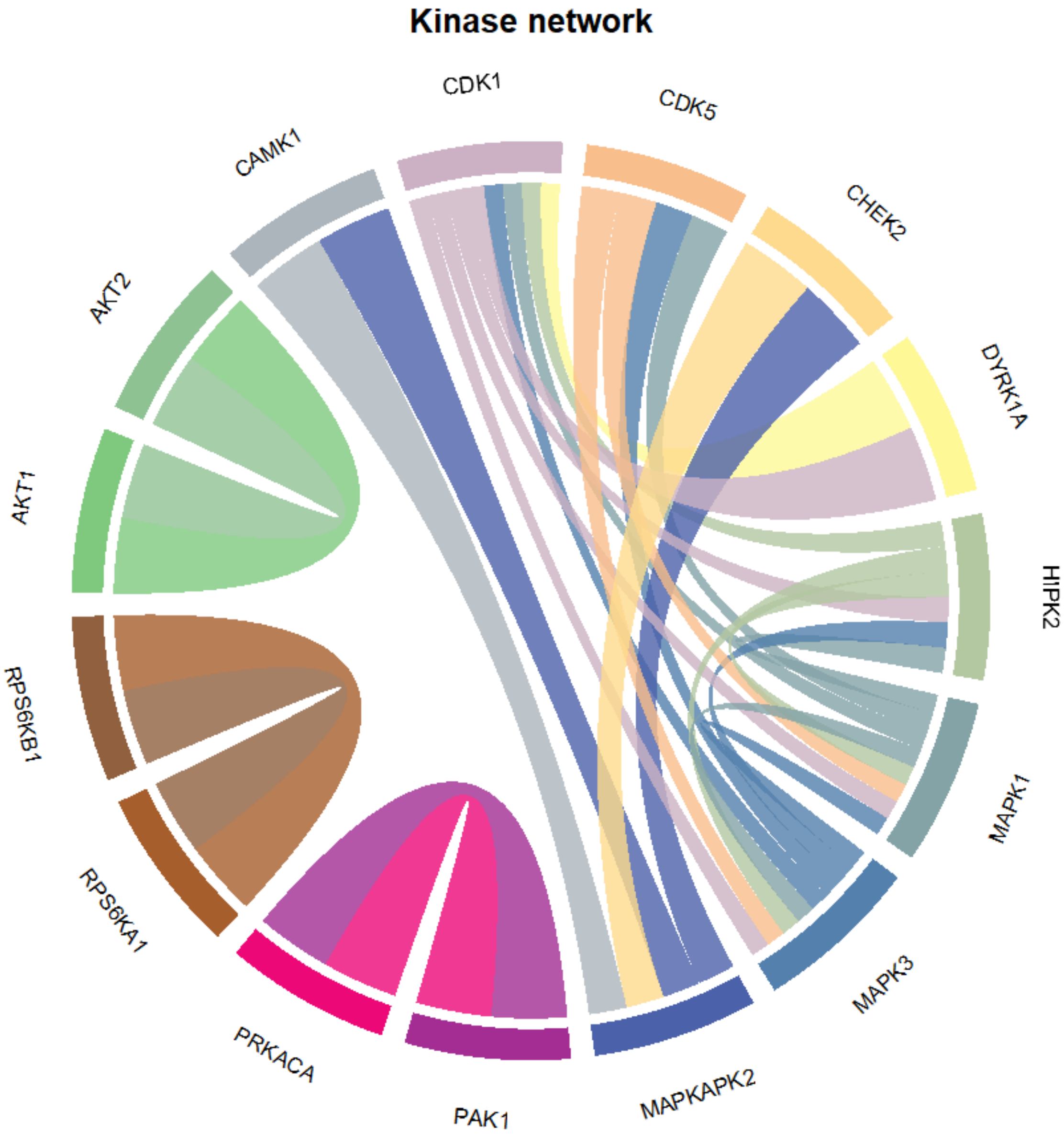

Figure S16. Interaction network of the inferred kinases based on the 183 altered phosphosites. Edges between nodes connect kinases that regulate common phosphosites.

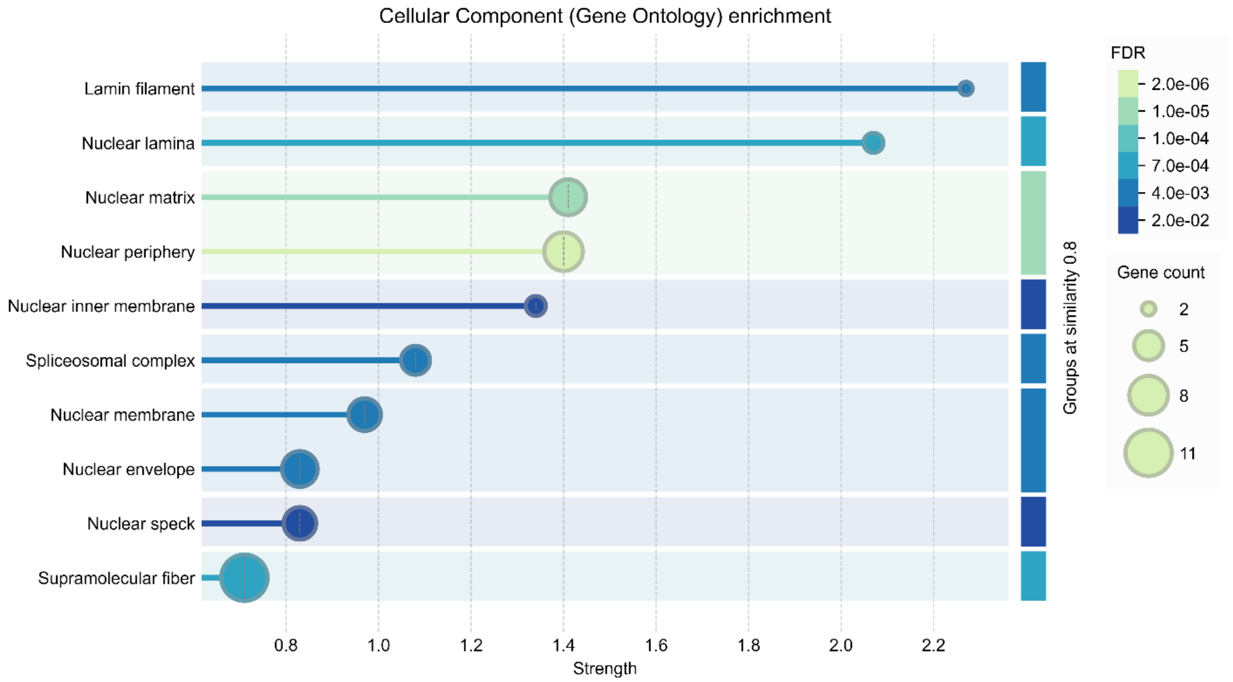

Figure S17. Visualization of functional enrichment analysis in STRING for GOCC terms of 44 phosphoproteins with potential alteration-specific phosphosites. The size of the dots corresponds to the number of genes associated with the term, and the color represents the FDR.

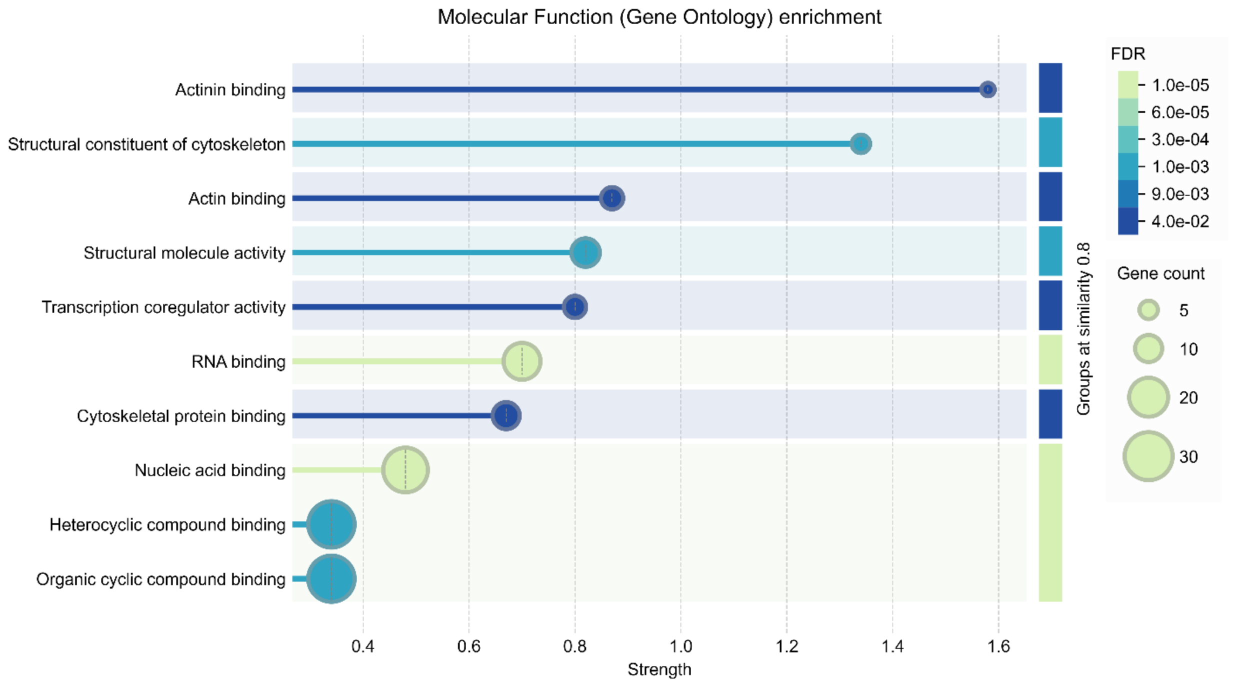

Figure S18. Visualization of functional enrichment analysis in STRING for GOMF terms of 44 phosphoproteins with potential alteration-specific phosphosites. The size of the dots corresponds to the number of genes associated with the term, and the color represents the FDR.

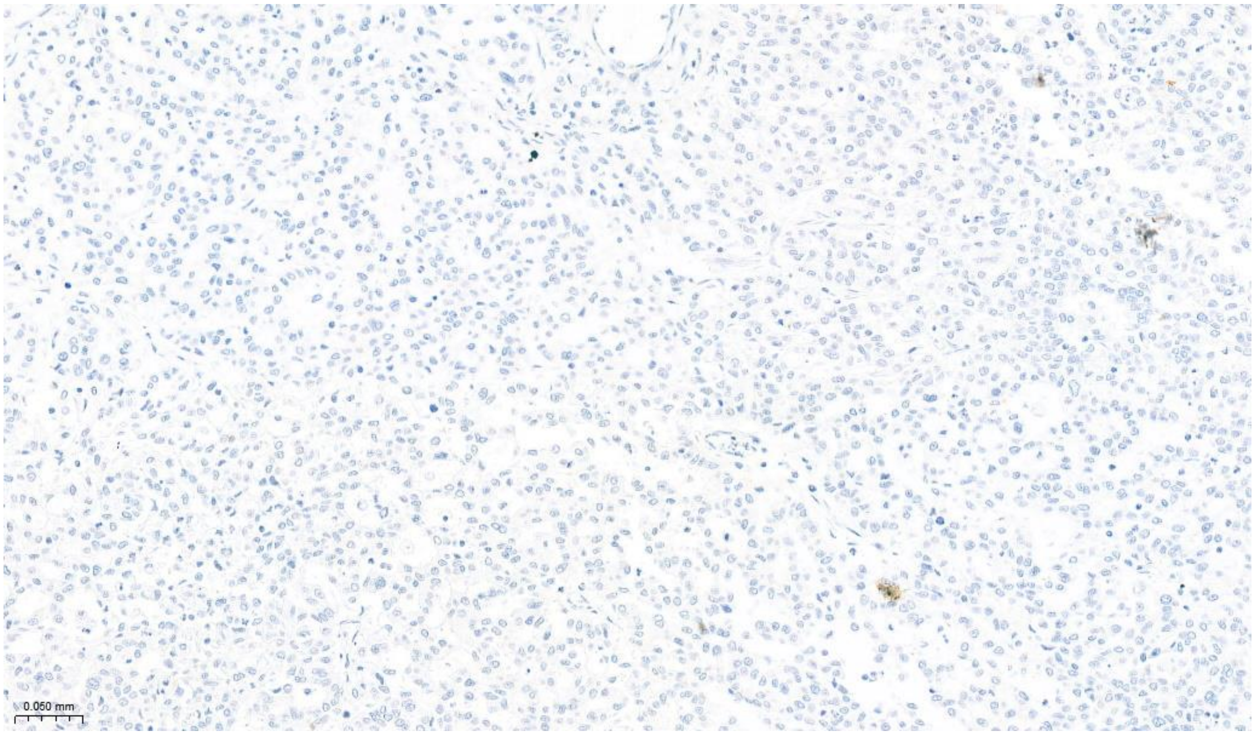

Figure S19. Immunohistochemistry staining of phospho-CDK2 (Thr160) antibody to assess CDK2 activity in EML4–ALK-rearranged LUAD tumor. Scale bar: 50 µm.

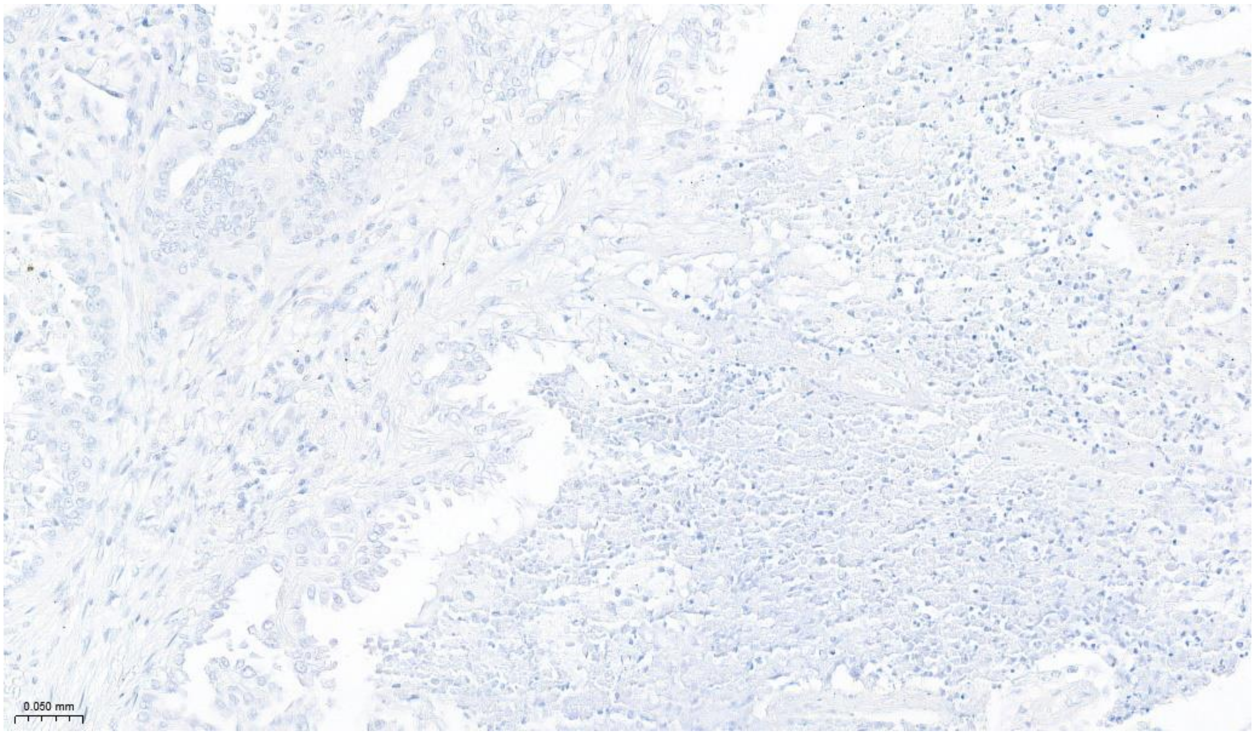

Figure S20. Isotype control for immunohistochemistry analysis. Scale bar: 50 µm.
